# Atrial Fibrosis Hampers Non-invasive Localization of Atrial Ectopic Foci From Multi-Electrode Signals: A 3D Simulation Study

**DOI:** 10.3389/fphys.2018.00404

**Published:** 2018-05-18

**Authors:** Eduardo Jorge Godoy, Miguel Lozano, Ignacio García-Fernández, Ana Ferrer-Albero, Rob MacLeod, Javier Saiz, Rafael Sebastian

**Affiliations:** ^1^Computational Multiscale Simulation Lab, Department of Computer Science, Universitat de Valencia, Valencia, Spain; ^2^Department of Bioengineering, Scientific Computing and Imaging Institute, University of Utah, Salt Lake City, UT, United States; ^3^Centro de Investigación en Bioingeniería, Universitat Politécnica de Valencia, Valencia, Spain

**Keywords:** atrial tachycardia, body surface potential map, structural remodeling, ectopic focus location, optimal electrode location, machine-learning

## Abstract

**Introduction:** Focal atrial tachycardia is commonly treated by radio frequency ablation with an acceptable long-term success. Although the location of ectopic foci tends to appear in specific hot-spots, they can be located virtually in any atrial region. Multi-electrode surface ECG systems allow acquiring dense body surface potential maps (BSPM) for non-invasive therapy planning of cardiac arrhythmia. However, the activation of the atria could be affected by fibrosis and therefore biomarkers based on BSPM need to take these effects into account. We aim to analyze the effect of fibrosis on a BSPM derived index, and its potential application to predict the location of ectopic foci in the atria.

**Methodology:** We have developed a 3D atrial model that includes 5 distributions of patchy fibrosis in the left atrium at 5 different stages. Each stage corresponds to a different amount of fibrosis that ranges from 2 to 40%. The 25 resulting 3D models were used for simulation of Focal Atrial Tachycardia (FAT), triggered from 19 different locations described in clinical studies. BSPM were obtained for all simulations, and the body surface potential integral maps (BSPiM) were calculated to describe atrial activations. A machine learning (ML) pipeline using a supervised learning model and support vector machine was developed to learn the BSPM patterns of each of the 475 activation sequences and relate them to the origin of the FAT source.

**Results:** Activation maps for stages with more than 15% of fibrosis were greatly affected, producing conduction blocks and delays in propagation. BSPiMs did not always cluster into non-overlapped groups since BSPiMs were highly altered by the conduction blocks. From stage 3 (15% fibrosis) the BSPiMs showed differences for ectopic beats placed around the area of the pulmonary veins. Classification results were mostly above 84% for all the configurations studied when a large enough number of electrodes were used to map the torso. However, the presence of fibrosis increases the area of the ectopic focus location and therefore decreases the utility for the electrophysiologist.

**Conclusions:** The results indicate that the proposed ML pipeline is a promising methodology for non-invasive ectopic foci localization from BSPM signal even when fibrosis is present.

## 1. Introduction

Focal atrial tachycardia (FAT) is a supraventricular tachycardia that triggers fast atrial rhythms from a location outside the sinoatrial node (Saoudi et al., 2001). FAT is commonly treated by radiofrequency ablation (RFA) with a high long-term success rate. The catheter ablation treatment targets the arrhythmogenic electrical drivers and terminates them by localized energy delivery. The end point of catheter based ablation is to eliminate the triggers with the least amount of ablation necessary (Santangeli and Marchlinski, 2017). In the case of FAT, the localization of those drivers tends to appear in specific hot-spots (Kistler et al., 2006), for example the pulmonary veins (PV) ostia are the most common sites of origin of focal tachycardias within the left atrium (LA) (Hoffmann et al., 2002), however they can be found virtually in any region of the atria, which makes their treatment difficult. The prevalence and distribution of focal triggers in persistent and long-standing atrial fibrillation has also been studied, showing a higher prevalence in the pulmonary veins for most groups, although non-PV triggers were observed in 11% of the cases (Santangeli et al., 2016). Electro-anatomical 3D mapping (EAM) is the standard technique used to obtain detailed intra-atrial activation sequences with the aim of bounding the source of the tachycardia (Bhakta and Miller, 2008; Santangeli and Marchlinski, 2017; Santoro et al., 2018).

Some factors might coexist with the tachycardia such as heart disease, hypertension or diabetes that could induce a structural remodeling process and the proliferation of fibrosis. Atrial fibrosis increases also with age and grows in conjunction with cardiomyopathy and heart failure (Go et al., 2001). Fibrosis has been linked to an increased incidence of rhythm disturbances via interaction with healthy tissue (Spach and Boineau, 1997). In addition, fibrosis distribution and density have been proposed as a predictor of recurrence in patients after a pulmonary vein isolation procedure by RFA (Oakes et al., 2009).

Detailed biophysical and anatomical models of the atria and torso have been successfully employed to reproduce complex electrical activation patterns observed in experiments and clinics (Trayanova and Boyle, 2014). Most of these studies, however, have focused on understanding the mechanisms that maintain certain types of arrhythmia such as atrial fibrillation (Zhao et al., 2015; Guillem et al., 2016), or spiral wave dynamics (Jalife, 2011), rather than providing solutions to tailor their treatment. In the last years, the analysis of arrhythmic patterns from non-invasive recordings such as multi-electrode surface ECGs using multi-scale biophysical models is starting to draw some attention as an alternative to EAMs (Shah et al., 2013; Giffard-Roisin et al., 2016).

The use of multi-electrode surface ECG systems allows for dense body surface potential maps (BSPM) with the aim of improving diagnosis of cardiac arrhythmia. A few attempts have been already carried out in clinics to relate BSPM-derived indices with atrial arrhythmic events induced artificially from an intracardiac catheter (Shah et al., 2013). From the modeling perspective, algorithms have been developed, mainly based on decision trees, to help identify the source of FAT from BSPM data (Kistler et al., 2006). In most of the previous studies, the presence of fibrosis has been neglected or not considered in the models. Ignoring the effects of fibrosis is a clear limitation since current-resistant fibrotic tissue affects the activation patterns.

In this study, we aim to predict the triggering site of a FAT using only BSPM data to help electrophysiologists pre- and intra-operatively, reducing the time to find and ablate the source. To achieve this goal, we have to be able to relate a BSPM-derived index with the source of a FAT even in the face of fibrosis patches that are present in different distributions and densities. In addition, we set out to ascertain the effect of fibrosis on the BSPM-derived indices. The proposed method uses machine learning techniques to develop a prediction pipeline that should be able to learn the relationship between BSPMs and ectopic foci location. We trained this system with a simulation database, generated by means of a detailed biophysical model of 3D human atria, in which we have control of the input parameters, and can simulate the desired scenarios.

## 2. Materials and methods

### 2.1. Anatomical model

**Figure 1:**
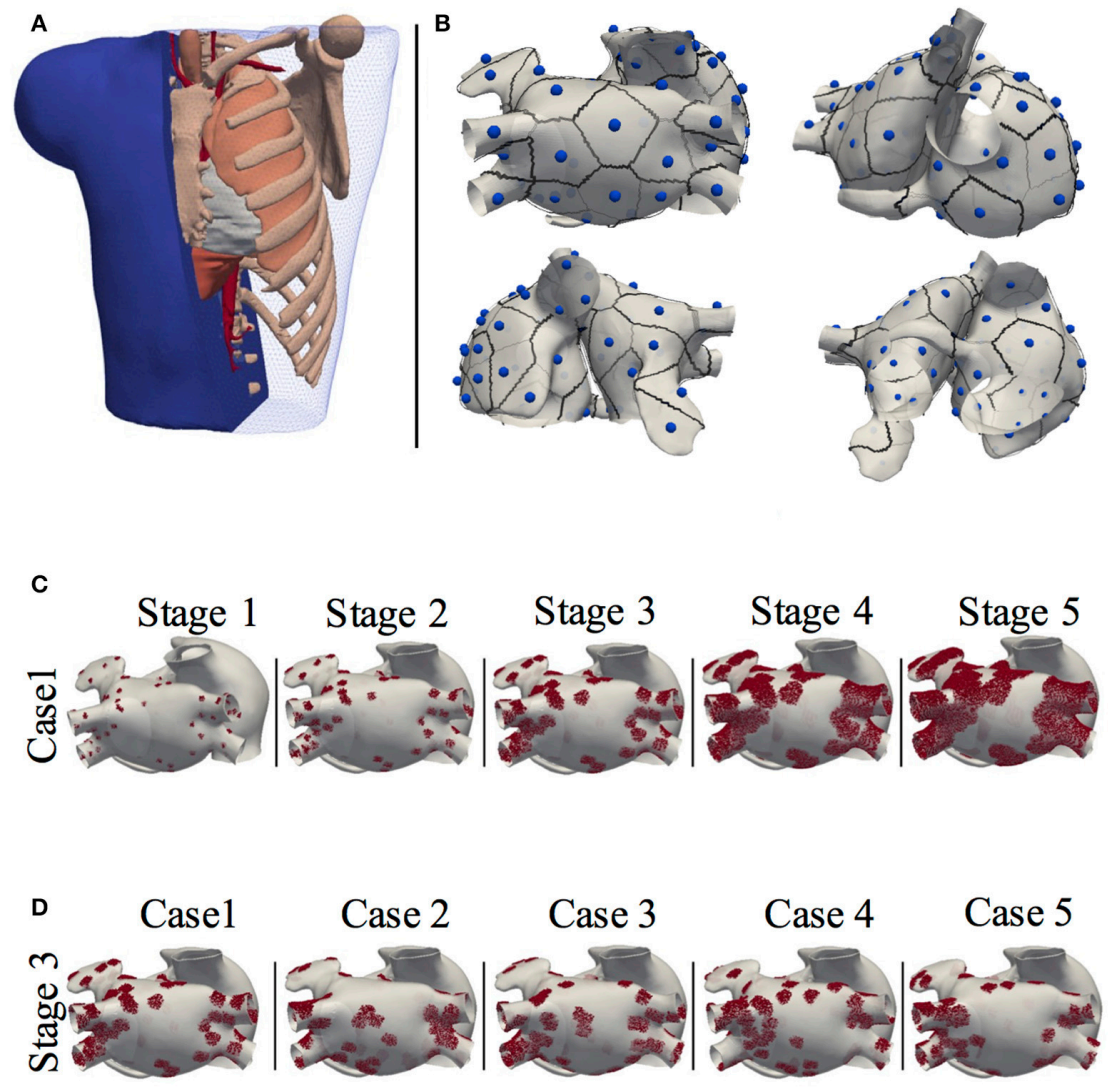
Model of the human atria and torso. **(A)** 3D torso model of tetrahedral elements, made up of lungs, bones, liver, ventricles, blood and general torso; **(B)** Localization of the 57 ectopic foci on the atria, together with their region of influence or patch. **(C)** The 3D atria including stages of patchy fibrosis. Case 1 shown for the 5 stages of fibrosis; **(D)** Stage 3 shown for the 5 different cases.

The 3D geometrical model of the atria and torso used in this study was previously developed (Ferrer et al., 2015). It consists of a highly detailed 3D geometric model of the atria (754,893 nodes and 515,005 hexahedral elements with a homogeneous resolution of 300 μm) coupled to a torso model (254,976 nodes and 1.5 M tetrahedral elements) made up of lungs, bones, liver, ventricles, blood, and general torso, see Figure 1A. The atrial model includes specific fiber orientations in 21 different atrial regions, heterogeneous tissue conductivity and anisotropy ratios and heterogeneous cellular properties adjusted following the model by Ferrer-Albero et al. (2017) and summarized in Table 1.

**Table 1:** Parameters used to reproduce cellular and tissue atrial heterogeneity.

**Prop**.	**RA**	**PM**	**CT/BB**	**TVR**	**RAA**	**LA**	**FO**	**MVR**	**LAA**	**PV**	**CS**
*g*_*to*_	1.00	1.00	1.00	1.00	0.68	1.00	1.00	1.00	0.68	1.00	1.00
*g*_*CaL*_	1.00	1.00	1.67	0.67	1.00	1.00	1.00	0.67	1.00	1.00	1.00
*g*_*Kr*_	1.00	1.00	1.00	1.53	1.00	1.60	1.60	2.44	1.60	2.20	1.60
σ_*l*_	0.003	0.0075	0.0085	0.003	0.003	0.003	0.000	0.003	0.003	0.0017	0.006
σ_*t*_/σ_*l*_	0.35	0.15	0.15	0.35	0.35	0.35	1.00	0.35	0.35	0.5	0.5
*CV*_*L*_	63.3	115.4	100.0	63.3	63.3	63.3	0.0	62.9	63.3	75.0	97.2

On the base atrial model, we included 5 different random distributions of patchy fibrosis in the left atrium. To define the fibrotic regions we included 50 seeds distributed among the following LA regions: Pulmonary veins, coronary sinus, ring fosa ovalis, and posterior LA wall in different proportions as in Zhao et al. (2013). Following, we grew each of the seeds using the region growing technique, so that fibrosis expanded forming patches. However, to avoid unrealistic perfect spherical fibrotic regions, we randomly reassign patchy elements back to healthy in the contours of the growing patch in each iteration, forming random fibrotic tissue shapes that might include surviving healthy tissue surrounded by fibrosis.

The fibrotic areas were grown according to the Utah classification (Oakes et al., 2009; Daccarett et al., 2011), that defines up to four levels of LA remodeling (quartiles) of fibrosis associated to the ratio of fibrosis to atrial volume [Utah stage I: < 8.1% (Q1); Utah Stage II: < 16% (Q2); Utah Stage III: < 21% (Q3) ; Utah Stage IV: >21% (Q4)]. Therefore, from the initial 5 fibrotic distributions, fibrosis was grown, as describe above, generating a total of 25 3D models. Note that for each of the 5 initial distributions we developed two models for quartile Q4 that we called Stage IV and Stage V. Figure 1C shows the atrial model for one of the 5 fibrotic distributions (Case 1) with fibrotic patches at each of the stages, and Figure 1D shows the atrial model for Stage 3, and for the 5 cases of fibrotic distribution. The figure shows how fibrotic areas can have any shape and include small islands of surviving tissue within the patches. Figure S2 shows the 25 distributions of patchy fibrosis used.

### 2.2. Electrophysiology modeling

For the electrophysiology simulations, we considered electrophysiological cellular heterogeneity in 10 different regions by adjusting *I*_*to*_, *I*_*CaL*_, and *I*_*Kr*_ in the Courtemanche-Ramirez-Nattel (CRN) ionic model (Courtemanche et al., 1998), plus the well-established fibroblast cell model by MacCannell et al. (2007) coupled to the CRN model. The tissue conductivities for each region defined together with their anisotropy ratios were obtained from Ferrer-Albero et al. (2017) and summarized in Table 1. The first three rows are the multiplicative factors used for the maximum conductance of three (*g*_*to*_, *g*_*CaL*_, and *g*_*Kr*_) ion channels with respect to the base Courtemanche-Ramirez-Nattel (CRN) ionic model (Courtemanche et al., 1998), and the next three rows are the longitudinal conductivity (σ_*l*_), the ratio between the transverse and longitudinal conductivities (σ_*t*_/σ_*l*_), and the longitudinal conduction velocity (*CV*_*L*_).

Propagation of excitation in the atria was calculated solving the reaction-diffusion, mono-domain equations, Equations (1) and (2), given by Geselowitz and Miller (1983) with the finite element method using the operator splitting numerical scheme by ELVIRA software (Heidenreich et al., 2010),

(1) $$\begin{array}{l} \nabla .(\text{D}\nabla V)=C_{m}.\frac{\partial V}{\partial t}+I_{ion}\;in\;\Omega_{H} \end{array}$$

(2) $$\begin{array}{l} \text{n}.(\text{D}\nabla V)=0\;in\;\partial \Omega_{H} \end{array}$$

where **D** is the equivalent conductivity tensor, *I*_*ion*_ is the transmembrane ionic current that depends on the cellular model, *C*_*m*_ is the membrane capacitance and Ω_*H*_ is the heart domain.

The extracellular potentials *V*_*e*_ in the torso model coupled to the atria were calculated using an approximation of the bidomain model. The first step was to interpolate the transmembrane potentials (*V*) obtained in the hexahedral mesh nodes of the atria to the tetrahedral torso mesh, overlapping the atrial region. The second step corresponds to the calculation of the extracellular potential by solving the passive term of the bidomain model (Keller et al., 2010), Equation (3), using the finite element method with Dirichlet and Neumann boundary conditions (Weber et al., 2011).

(3) $$\begin{array}{l} \nabla .(\text{D}\nabla V)+\nabla .(({\text{D}}_{i}+{\text{D}}_{e})\nabla V_{e})=0 \end{array}$$

where **D_i_** and **D_e_** are the volume-average conductivity tensors of the intra and extracellular domains (Niederer et al., 2011).

#### 2.2.1. Analysis of cell coupling

In order to analyze the coupling of the different cell models with the fibrosis, we performed a preliminary analysis on a 3D slab of tissue that combined healthy atrial tissue and fibrotic tissue to assess: (i) changes in action potential duration (APD), and (ii) conduction velocities. The 3D slab dimensions were 50 × 50 × 0.3 mm and were built with voxel elements of 300μm in size. Several configurations of the fibrotic tissue were designed to evaluate: (i) the minimum amount of fibrosis required to produce a conduction block in a wavefront advancing perpendicular to the line of fibrosis; (ii) the minimum conduction channel (healthy tissue surrounded by fibroblast) necessary to allow the propagation of the electrical impulse.

### 2.3. Simulation of BSPM during focal atrial tachycardia

The 3D atrial models were prepared for simulation of FAT from 19 different triggering locations (including sinus rhythm) following clinical studies (SippensGroenewegen et al., 2004). See Figure S1 for a graphical description of the 19 triggered locations selected out of the 57 initial locations included in Figure 1A. Triggering points were present in both the left and right atria, and were chosen to cover most of the atrial wall, in regions prone to elicit ectopic activity. Only in the case of the atrial model without fibrosis, did we consider an additional set of 38 extra triggering points in order to have more information on the healthy activation maps as in Ferrer-Albero et al. (2017). Figure 1B shows the 57 ectopic locations used. For a more detailed description, see Table S1, in which the different anatomical regions in the atria with simulated ectopic foci are shown. Figure S3 shows the LATs for all ectopic foci (Case 1), while Figure S4 shows their corresponding BSPiMs.

Before triggering the FAT, all 3D models were stabilized by simulating 20 heart beats in sinus rhythm with a basic cycle length (BCL) of 500 ms. This was necessary to homogenize the coupling of the different cellular populations and the fibrotic tissue, smoothing differences between neighboring regions. Next, simulations corresponding to each of the 19 ectopic beat locations were carried out for each fibrotic configuration which resulted in 475 simulations in the 3D atrial models with fibrosis, plus 57 simulations in the non-fibrotic model.

Body surface potential maps (BSPM) were obtained for all the simulations by calculating the extracellular potential at all the nodes of the torso surface. To obtain more realistic results, we added white Gaussian noise to simulate the effect of noise from muscles or other sources on the BSPM. An average P wave had a mean power of 0.003 mW (i.e., −55.2 dBW), and we added white Gaussian noise with a mean power of 0.001 mW (i.e., −60.0 dBW), yielding an approximate power ratio of 3 and S/N ratio of 4.8 dB. Afterward, we filtered the signal using a Savitzky-Golay smoothing filter that minimizes the least-square error in fitting a polynomial to frames of noisy data. It is optimal in the sense that performs much better than the standard FIR filters, which tend to filter out a significant portion of high frequency content along with the noise (Orfanidis, 1995).

Once all the filtered noisy BSPMs were obtained, the body surface potential integral maps (BSPiM) were calculated, as described in SippensGroenewegen et al. (2004). As a result, for each ectopic focus simulated we summarized each P-wave signal on the torso surface into a single value obtained from integrating the corresponding P-waves at each torso point, which resulted in the BSPiM. The integration of the BSPiM is equivalent to the average of the electrical cardiac vector over time, and therefore describes how the depolarization wavefront advances during the activation. The BSPiMs were all normalized in the range [−1,1].

### 2.4. Machine learning pipeline

**Figure 2:**
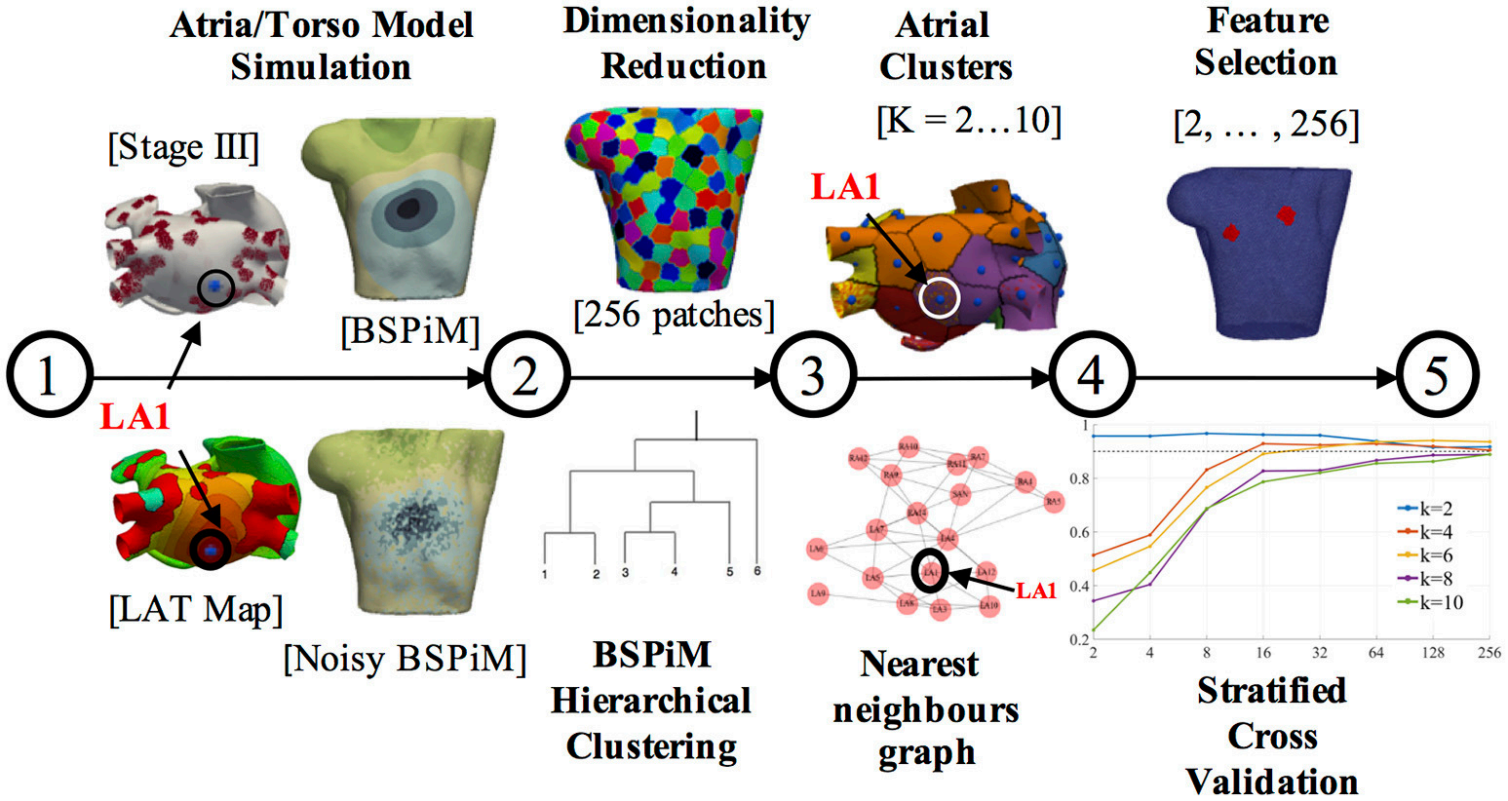
Machine learning pipeline. The pipeline consists of 5 steps. Steps 1 and 2 are performed for each ectopic focus and fibrosis configuration. Steps 3, 4, and 5 are the training and validation phases and carried out with all the simulation results. The different torso and atrium figures shown correspond to ectopic focus LA1, located on the LA posterior wall.

The simulation of ectopic foci allowed us to obtain a data set formed by the ectopic locations on the atria (3D points) and the corresponding BSPiMs they produced on the torso. Using this data set, machine learning techniques could be applied to establish a relationship between the location of an ectopic focus and the BSPiM map it produced. Following this idea, we designed the pipeline shown in Figure 2 to solve the ectopic localization problem. This pipeline had five main steps. Firstly, we performed the simulations for every ectopic focus on the atria and obtained the corresponding BSPiM. Next, we performed a dimensionality reduction on the BSPiMs, followed by a clustering of BSPiMs. We validated all the clusters obtained to evaluate their *quality*. Finally, we performed a stratified cross-validation, to assess the classification accuracy of the ectopic foci into the different clusters defined. Note that in this context, a cluster of ectopic foci corresponds to a region on the atria.

The first two steps in the pipeline can be considered as a pre-processing stage, and they were necessary to generate and reduce the resolution of the raw BSPiMs, which originally had more samples than those in a real clinical setting. In this pipeline, we used two datasets, one corresponding to the 57 ectopic foci simulated without fibrosis, which contained 57 activation sequences. The second data set corresponded to the 25 fibrotic configurations × 19 triggering locations containing 475 activation sequences.

**Step 1 - Atria/Torso Model simulation:** In this step the simulation of ectopic foci was performed by solving Equation (1) in the atria domain and Equation (3) in the torso domain, which generated the BSPMs. After adding noise to the P-wave signals for all the computational nodes, we filtered the resulting signals. We performed a trapezoidal integration of the noisy P-wave to obtain the BSPiMs. As an example, Figure 2 (step 1) contains the activation patterns produced by the ectopic focus LA1 (located on the left atrium (LA) posterior wall), for a configuration with fibrosis remodeling in stage III, together with the corresponding BSPiM with and without noise (frontal views) for the same ectopic focus.

**Step 2 - Dimension reduction and BSPiM clustering:** This step corresponded to an unsupervised learning phase to classify the filtered noisy BSPiMs that result from the activation of the different ectopic foci (457 + 57 simulations).

The computational torso model used in this pipeline had 14,157 surface nodes, and therefore provided BSPiMs with that resolution. Before the clustering phase, these dense BSPiM data sets were reduced to a more feasible clinical scenario, since current BSPM vest system technologies allow a maximum of about 256 electrodes placed on the torso of a patient (Shah et al., 2013). Therefore, the dimension of each input data set of filtered noisy BSPiM, was homogeneously reduced to a maximum of 256 nodes, (i.e., features) (see Figure 2, step 2, top). To select the nodes, we divided the torso domain into 256 equally-sized patches (55 nodes per patch in average), and choose randomly one of the nodes in each patch to represent the whole region. This is a sensible approach since in a clinical BSPM acquisition system the exact location and spacing of the mapping electrodes may not be perfectly preserved across patients.

The unsupervised clustering of the 256-dimensional BSPiM patterns was performed using hierarchical/agglomerative clustering. One of the benefits of hierarchical clustering is that one does not need to know in advance the number of clusters *K* in the dataset, assigning each sample to its natural class. We used the Ward et al. variance minimization algorithm (Ward, 1963). We started with a single cluster for each sample and iterated by finding, at each step, the pair of clusters that, after merging, produced the minimum increase in the total within-cluster variance.

We also used dendrograms for visualization in the form of trees showing the order and distances of merges during the hierarchical clustering process. We repeated this phase imposing a distance limit in the algorithm, obtaining K clusters ranging from 2 to 10 clusters (see Figure 2, step 2, down).

**Step 3 - Clusters validation on the atrial surface:** The assignation of every BSPiMs to a cluster induced a clustering on the set of ectopic foci that produced the corresponding BSPiM; if a certain BSPiM was assigned a label *j* by the clustering algorithm, then we assigned the same label to the ectopic location that produced that BSPiM. Now, the question to resolve was whether the clustering that was mapped onto the atria had some geometrical meaning to identify the location of the ectopic focus. To analyze this, we associated to each ectopic focus a region or patch formed by the points in the atria closer to that focus than to any other. From the clustering induced in the atria, we could associate also a patch to every BSPiM cluster, built as the union of the patches of the associated ectopic foci. In Figure 2 (step 3, top) we show an example of the clustering generated on the atria surface. Each atrial patch associated to an ectopic focus has a color that corresponds to its class. In the example, ectopic LA1 (for *K* = 6 groups and fibrosis Stage III) was associated to the purple class.

Recall that our goal was to build a system that takes a measured BSPiM and predicts the location of the ectopic focus that generated it or, at least, a region where it could be located. Thus, the ideal situation would be that two BSPiM that are in the same cluster are the result of the same ectopic focus or, at least, of two ectopic foci that are in nearby patches in the atria. By contrast, an adverse situation would happen when similar BSPiM would be clustered together due to their similarity, but the ectopic foci that generated them were distributed along the atria and did not form a connected region on the surface. We defined a well formed region/cluster as a union of ectopic patches that was connected and only included ectopic foci from the same cluster.

Thus, we sought to assess the quality of the patches formed from the BSPiM clustering. To be able to verify this requirement we created an ectopic graph, where the nodes represent the ectopic foci and are connected by an edge if their associated patches share a border. The geodesic distances on the atria were obtained by a Fast Marching algorithm (Kimmel and Sethian, 1996). According to the ectopic graph created, a well formed patch could be identified as a connected subgraph containing all the ectopic foci of a class (see Figure 2, step 3, down). In this way, we reduced the problem of deciding if a cluster is well formed to a connectivity test on the associated subgraph.

It is important to note that for each ectopic location we had 25 simulations (5 different fibrosis configurations × 5 different fibrosis stages) with differing fibrosis distribution. An ideal result would be that all the BSPiM simulations produced by the same ectopic focus end up in the same cluster, regardless of the fibrosis configuration or stage. However, due to the changes in LAT due to fibrosis such a result would have been highly unlikely.

Considering *K* as the number of clusters, we define the *persistence* of an ectopic location *x* as the number of different clusters that contains ectopic foci located at *x*, divided by the number of clusters, *K*. The best situation for an ectopic is produced when it only appears in one class (fully persistent), with a persistence value of 1/*K*, while the worst situation occurs when it appears in all the classes, with a persistence value of 1. Ectopic locations with a poor value of persistence would indicate that several cluster patches overlap on that ectopic patch. This situation will be represented in our figures with regions that have spots of more than one color.

It is noteworthy, however, that a poor persistence value does not necessarily lead to a bad prediction situation. Although an ectopic location is in, say, three clusters, if the three clusters are well formed the system will still be able to indicate a meaningful region for the ectopic when a BSPiM is processed.

**Step 4 - Feature selection:** In addition to the dimensionality reduction carried out before the clustering step, we performed a feature selection step, in this case to select the best features among the 256 (see Figure 2, step 4, top). The reason for this further selection is that in many clinical procedures the number of available electrodes is far below 256. We wished to establish the minimum number of electrodes necessary to build a successful prediction system and to determine what their optimal locations are.

In our context, we will consider that a feature (representing an electrode location) is less relevant than another when its value is independent of the classification of the sample, from a probabilistic point of view. We performed a hypothesis contrast on the data set to assess the dependence of each component of the BSPiMs data with the class distribution of the samples. Given a feature, we consider its value and the class of the samples in the data set as random variables. Using the chi-square (χ^2^) test, if a small *p*-value is found for a given feature, it shows statistical evidence that the value of that feature is not independent from the class of the sample. Then, we keep the *N* best features and disregard those that are most likely to be independent of the class label. This process is repeated for *N* = 2, 4, 8, 16, 32, 64, 128, 256, which permits us to compare the performance of the system as a function of the final number of features selected.

**Step 5 - Ectopic foci Classification:** As a result of the cluster validation process, the generated clusters can be viewed as groups of patches on the atrial surface that relate to BSPiMs patterns. We trained a classifier able to classify any BSPiM into one of the clusters defined, that would point to a patch group [atrial region(s)]. For each number of clusters (*K* = 2,…10), we constructed a supervised learning model using a support vector machine (SVM) using the implementation in Pedregosa et al. (2011) and Buitinck et al. (2013). The SVM does the classification of the data finding the best hyperplane that separates all data points of one class from those of other classes. We used a 4-fold stratified cross validation process where different SVMs were trained to avoid over-fitting and to evaluate the prediction accuracy and the generalization level obtained. We adjusted the parameters (i.e., a regularization term) of a radial basis kernel for the decision function. Folds were selected so that the mean response value was approximately equal in all of them. Each fold contains the same proportion of ectopic foci from each cluster, and all the ectopic foci were tested. Having 4-folds leaves 75% of samples for training and 25% of samples for testing in each fold.

## 3. Results

### 3.1. Simulations on a 3D slab of tissue

**Figure 3:**
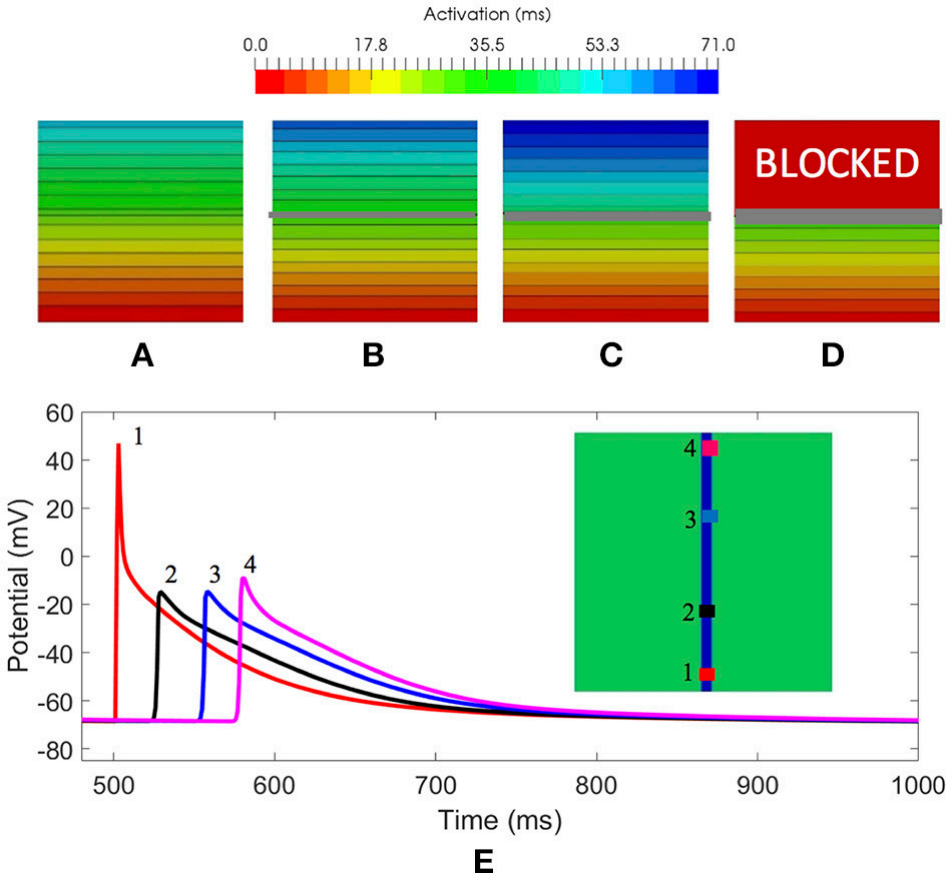
3D slabs of left atrial tissue including fibrosis barriers. **(A)** Shows a slab free of fibrosis with an activation time of 59 ms; **(B)** a slab with the same properties and a cross-wise barrier of one voxel element width (x-axis) of fibroblast cell model, taking a time for activation of 62 ms; **(C)** 3D slab including a cross-wise barrier of two voxel elements width (x-axis) of fibroblast cell model, taking a time for activation of 71 ms; **(D)** 3D slab including a barrier of three voxel elements width (x-axis) of fibrosis tissue model where the propagation front is blocked; **(E)** Study of the effect on the AP at 4 locations along a conduction channel of healthy atrial tissue with a width y-axis of 1.8 and 0.3 mm, on a slab of fibrosis tissue.

In a first evaluation, we analyzed the amount of fibrosis required to produce a conduction block. We included in the slab modeled as LA posterior wall tissue, a cross-wise line of fibroblast cells with different width (x-axis) to evaluate the effect on the propagation (see Figures 3A–D). We stimulated the slab with a cross-wise flat impulse that progressed activating the full width of the slab from bottom to top. Figure 3A shows a slab configuration free of fibrosis. The time required for the wavefront to travel the full size of the slab was 59 ms. Figure 3B, shows a slab that includes a cross-wise line of 0.3 mm modeled as fibroblast cells. The activation time was 62 ms, therefore the fibroblasts introduced a delay in the propagation wavefront of 3 ms (5% increase). When the fibrotic barrier was 0.6 mm, the wavefront took 71 ms to reach the top of the slab, that is a 12 ms delay (20% increase) with respect to the healthy configuration (see Figure 3C). When the barrier was increased to three voxels, i.e., 0.9 mm width, there was a conduction blockage (see Figure 3D). The results clearly show that the effect of fibrosis on the propagation wavefront in our model is not linear.

In a second evaluation, we inverted the configuration of the tissue types of the slab, that is, all tissue was modeled as fibroblast, except a conduction channel modeled as healthy atrial tissue, with different width {[1.4, 1.5, 1.8, 2.4, and 2.7 mm] (x-axis) × [0.3 mm] (z-axis)}. The goal was to determine the minimum width required for a conducting channel to propagate the electrical impulse when it is surrounded by fibrotic tissue. Figure 3E shows a slab of fibrosis tissue with a conduction channel of 1.8 × 0.3 mm, where we studied the AP at 4 locations distributed along the conduction channel. Table 2 summarizes the results obtained. Location 1 (trace in red color) shows the largest AP amplitude, since it is closer to the initial electrical shock delivered. Resting potential is elevated in all cases, but the effect is larger at locations 2, 3, and 4. Table 2 shows the AP values for locations 1 and 4, and for each channel width tested. The channel with a width of 1.2 mm produced a propagation block a few millimeters from the initial impulse. The intermediate channel (1.5 mm), propagated the signal, but the amplitude of the signal at the channel exit (location 4) was reduced by 54%, whereas in the wider channel (1.8 mm) it was reduced by 48%. All the *APDs*_90_ measured, for all locations in the conduction channel, were greatly reduced with respect to the APD for the original model. With respect to the delay, it was clear that the electrotonic coupling affected the AP rising time, with an increase of 40 ms in the propagation delay when channels were reduced to 1.5 mm. That delay equates to a decrease in conduction velocity from 0.85 to 0.50 m/s, due only to the effect of fibrosis coupling. Channels larger than 3.0 mm permitted a normal propagation of the signal in the center of the channel, with respect to conduction velocity and APD morphology.

**Table 2:** Properties of AP in the entrance and exit [locations (1)/(4)] of a conduction channel.

**Channel width**	**1.2 mm**	**1.5 mm**	**1.8 mm**	**2.4 mm**	**2.7 mm**	**>3.0 mm**
RMP (mV)	−63.6/–	−66.3/−65.8	−68.5/−68.0	−71.4/−71.0	−72.7/−72.2	−79.91
Peak value (mV)	39.4/–	43.0/−15.2	46.7/−9.0	50.7/−1.7	52.9/0.2	0.73
APD90 (ms)	65.0/–	104/122	127/168	153/189	161/195	252
Delay (4)-(1) (ms)	–	99.0	76.0	60.0	60.0	59.0

**Figure 4:**
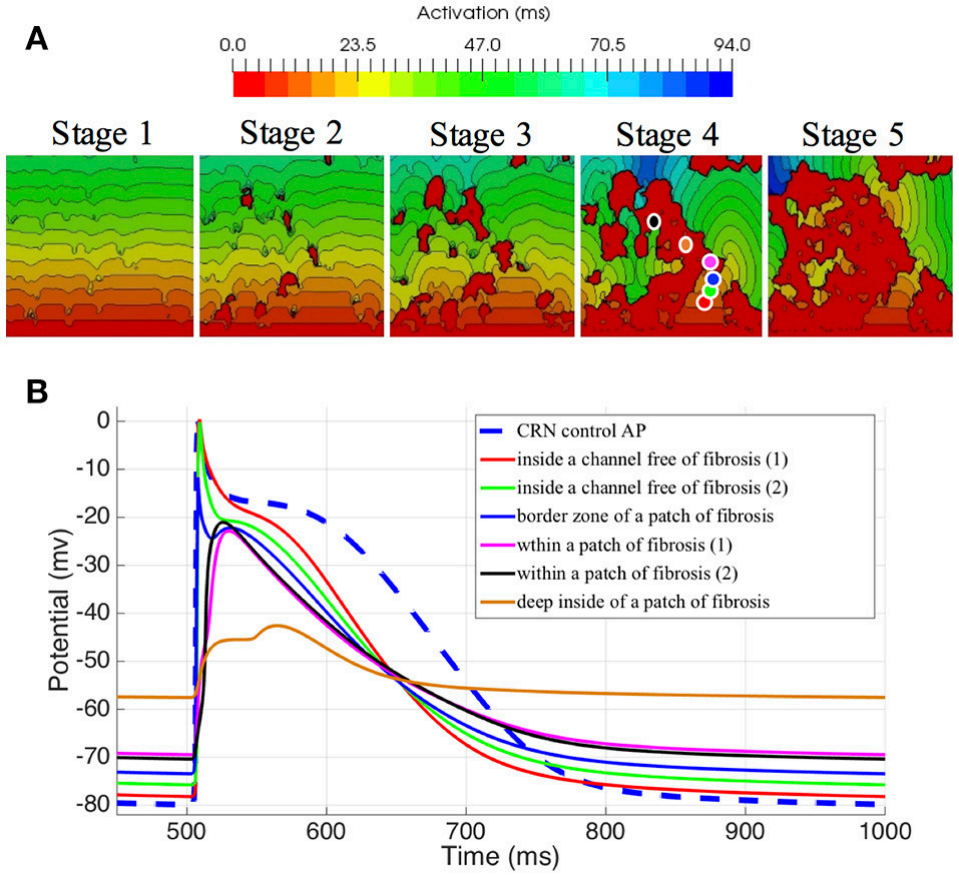
Electrophysiological effects on the Courtemanche-Ramirez-Nattel myocytes when coupling to the active formulation of MacCannell fibroblast model. **(A)** shows the local activation time map on 3D slabs of tissue that include fibrotic tissue in increasing amount (left to right). The color corresponds to the local activation time in ms. **(B)** shows the action potential on specific locations obtained from the 3D slab with fibrosis in Stage 4. Note that the color of each plot refers to the corresponding color-coded disc in the figure above.

Finally, using the same size and number of voxel elements, we analyzed the propagation changes as a function of the 5 different stages of patchy fibrosis, from less dense (Stage I) to more dense (Stage V) of fibrosis (see Figure 4A). The figure shows that as we increase the level of fibrosis, paths are formed by the fibrosis patches.

We also assessed the influence of the electrotonic interactions between atrial tissue myocytes and random shape and size fibrotic patches on the AP, APD, and the resting membrane potential (RMP); with that purpose, we used six probes located in different positions within the 3D slab of tissue with Stage IV of fibrosis. Those locations were selected to go across different areas of the slab, from a channel free of fibrosis to an area well inside a higher density zone of fibrosis, see Figure 4A, labeled Stage IV.

To appreciate the changes in the AP when coupling myocytes to fibroblasts, and the cell-to-cell electrotonic interaction, we overlapped the AP signals measured at each probe, together with an AP measured in a healthy myocyte, as control. Figure 4B together with Table 3 show those effects.

**Table 3:** Effects of fibroblast-myocyte interaction on the Action Potential (Shown for the Stage 4 of fibrosis).

**AP measurement/trace**	**Control**	**Red**	**Green**	**Blue**	**Magenta**	**Black**	**Orange**
RMP (mV)	−79.9	−78.2	−75.7	−73.4	−69.4	−70.3	−57.5
Peak value (mV)	0.73	0.39	−0.16	−11.67	−22.85	−21.04	−42.58
Amplitude (mV)	80.66	78.59	75.60	61.77	46.63	49.33	14.97
APD90 (ms)	252	209	212	224	219	218	200
APD50 (ms)	153	105	94	101	85	87	52

Electrophysiological effects on the Courtemanche-Ramirez-Nattel myocytes when coupling to the active formulation of MacCannell fibroblast model due to the cell-to-cell electrotonic interaction, caused a reduction of AP amplitude which can be appreciated with respect to the control trace as soon as the probe was near a more dense patchy fibrosis area. It produced a reduction of AP plateau, and a clear shortening of the myocyte APD90 with respect to the control AP and variable depending the location of the probe and the density of fibrosis. APD50 showed a more constant reduction of the APD as we went deep inside fibrotic areas showing a clear deformation of the AP profile. Also, it produced a prolonged repolarization of the AP compared to the uncoupled myocyte, and more significantly, fibroblasts had a higher resting membrane potential (RMP) and hence affected directly the myocyte RMP, which was constantly elevated (see Figure 4B).

### 3.2. Simulations on the atria-torso model

**Figure 5:**
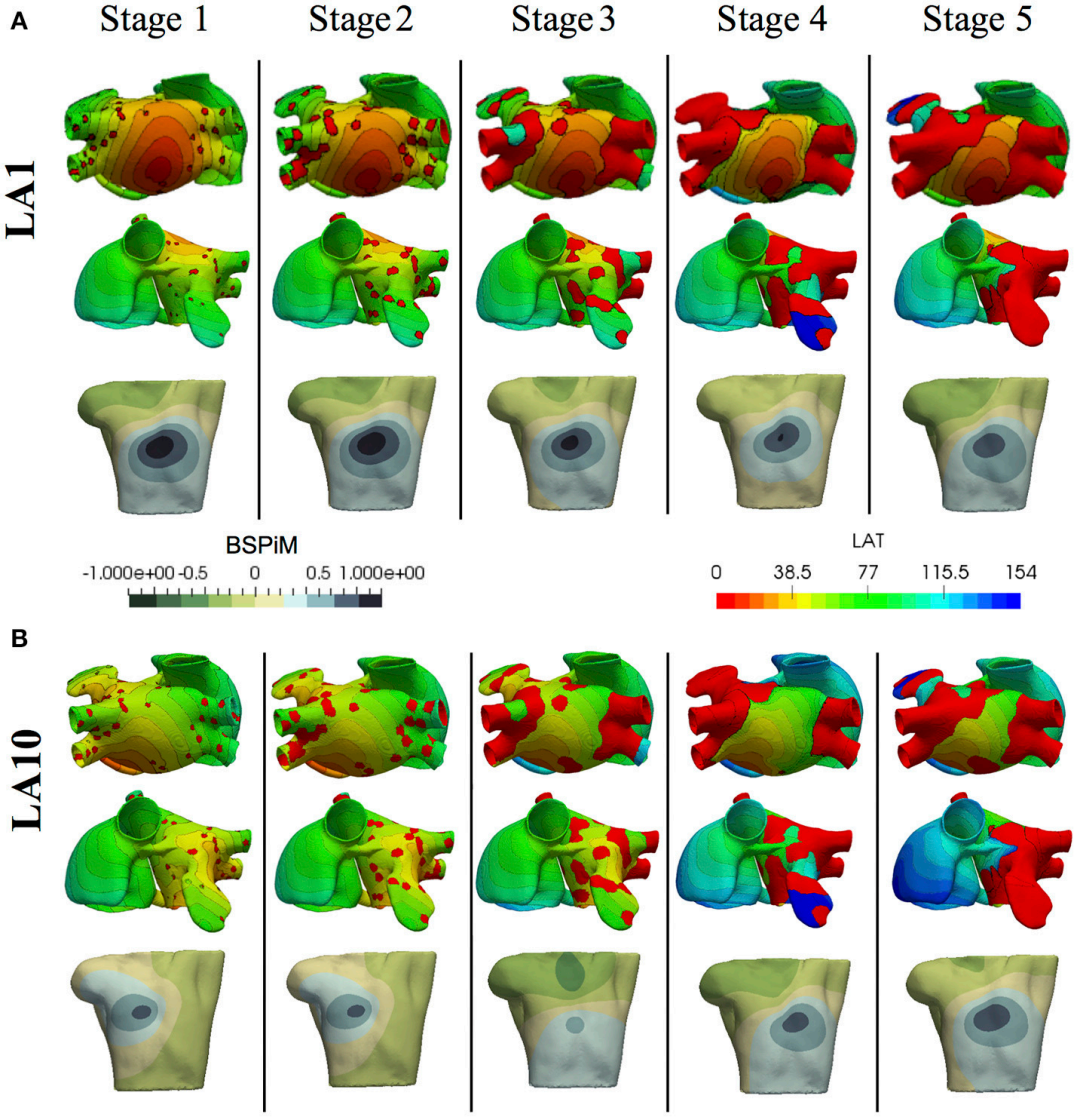
Local activation time (LAT) maps and BSPiMs generated by ectopic foci **(A)** LA1 and **(B)** LA10 for the 5 Stages of fibrosis and simulated for case 1, and the 5 stages of fibrosis.

Once the atrial simulations were completed, from the 475 simulations that included fibrosis, 54 of them were excluded because they did not trigger the FAT activity due to the proximity between the ectopic location and the fibrotic tissue. The excluded simulations were 5 from the Stage 3, 20 from the stage 4, and 29 from the stage 5. Activation maps for stages with more than 15% of fibrosis showed both conduction blockades and delays in propagation, fundamentally around the pulmonary veins, but also in some critical areas that prevented the electrical communication between both atria through standard pathways. As expected the larger changes in LAT maps were for stages 4 and 5 due to the conduction delays and blockades that changed the standard activation sequences (see Figure 5A and Figure S3). Figure 5 shows the LAT maps and BSPiM for two ectopic foci, LA1 located in the posterior LA wall and LA10 located in the area of the bicuspid mitral valve ring embraced by the coronary sinus. LA1 activation sequence was not greatly affected by the fibrosis along the different stages, except for the left atrial appendage and the pulmonary veins which did show very low voltage potentials that did not contribute to the activation sequence (Figure 5A, red regions). The corresponding BSPiM patterns showed little differences where the maximum values drifted slightly and the isochrones expanded. On the other side, LA10 showed important differences between stages for several reasons. Firstly, the communication between atria through Bachmann's Bundle was compromised, delaying the activation of the RA. Second, the activation of the LA appendage was also delayed as happened with LA1. As a result, LA10 BSPiM for stages 4 and 5 resembled LA1 more than LA10, which could hamper the training system that classifies ectopic foci.

#### 3.2.1. Clustering of BSPiM maps

For the clustering, learning, and classification of the ectopic foci we created six different subsets of BSPiMs named M0, …, M5. Table 4 summarizes the data included in each of those models. Model M0 includes the control data set without fibrosis, where FAT is triggered from 57 ectopic foci. The rest of models M1–M5 include fibrosis and are triggered from 19 locations. For instance, model M1 includes cases 1 to 5 with fibrosis stage 1 which makes 95 simulations. In some models simulations that did not propagate were excluded: 5 for M3, 20 for M4, and 29 for M5.

**Table 4:** Models created for the clustering, learning, and classification steps of the pipeline.

**Model**	**Fibrosis**	**Stages**	**# BSPiMs**
M0	No	–	57
M1	Yes	[1]	95
M2	Yes	[1,2]	190
M3	Yes	[1,.,3]	280
M4	Yes	[1,.,4]	355
M5	Yes	[1,.,5]	421

*The table shows the levels of fibrosis and the number of BSPiMs included in each model as data set*.

**Figure 6:**
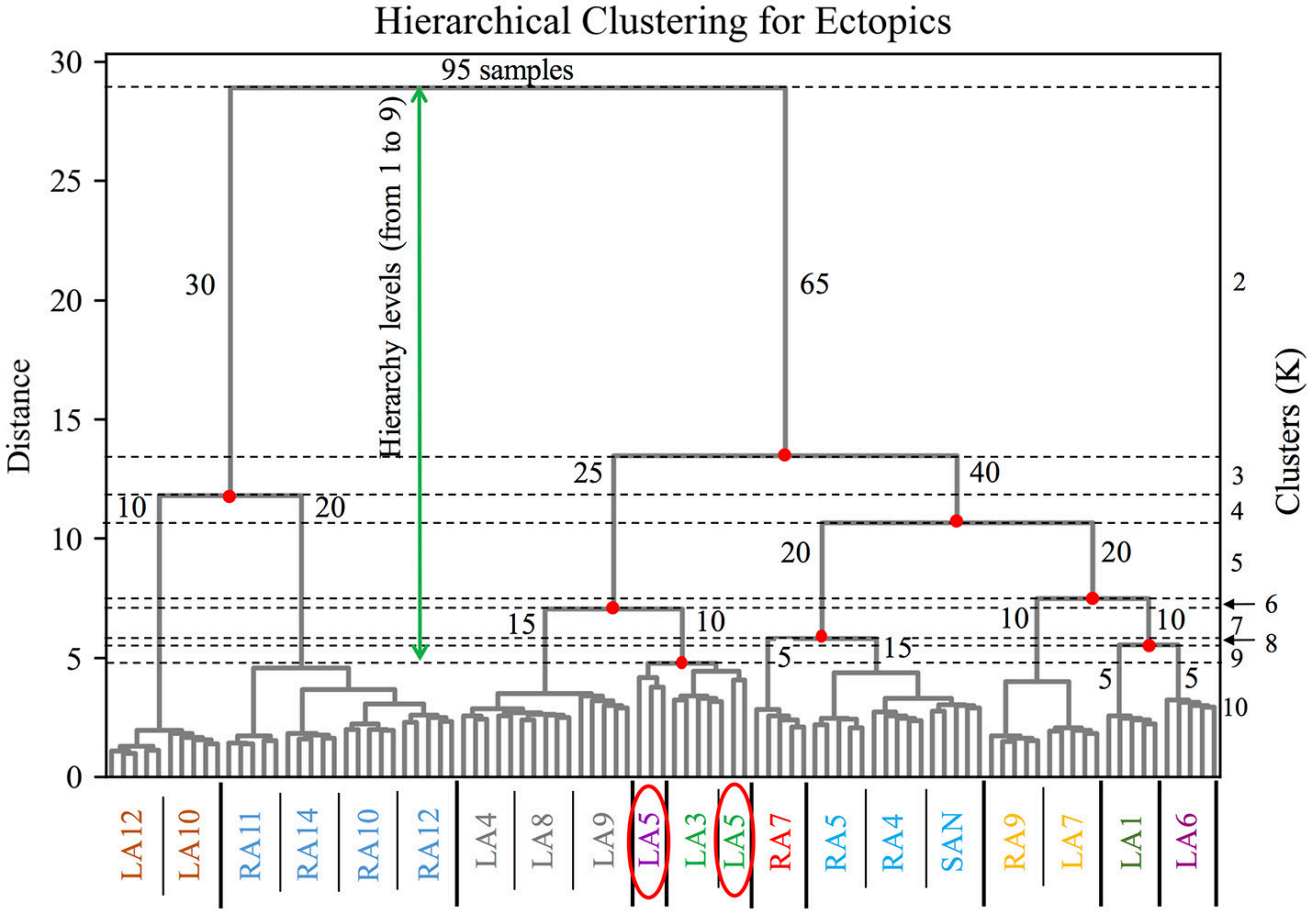
Dendrogram of the hierarchical/agglomerative clustering algorithm for the model M1. Hierarchical levels are indicated by the horizontal dotted lines from 1 to 9 (green arrow), the *y* axis on the left shows the distance between levels, and on the right the clusters being formed after merging and splitting samples (number of samples) at each level from *K* = 2 to *K* = 10. The *x* axis shows the color coded groups of ectopic labels separated by the thick vertical black line. The red ellipses show the particular case of the BSPiM produced by the ectopic LA5, being grouped in two different clusters, 3 samples to cluster 3, and 2 samples in cluster 4.

The clustering of the BSPiM maps obtained with the hierarchical/agglomerative clustering predicted a number of groups. Figure 6 shows, as an example of the performed hierarchical/agglomerative algorithm, the dendrograms for the model M1. The hierarchy levels (from 1 to 9) as a function of the separation distance (green arrow) are indicated by the horizontal dotted lines, and the clusters (K) being formed at each iteration, which are shown on the *y* right axis from *K* = 2 to *K* = 10. At each iteration the clusters being split have the smallest distance according to the Ward et. al. linkage algorithm (Ward, 1963).

Figure 6 shows how in its first iteration the linkage algorithm decided to split the 95 original samples of model M1 in two clusters (*K* = 2), one with 30 samples (number of samples per cluster shown in blue) and the other with 65 samples, with a large separation distance. In the second iteration, the cluster with 65 samples was divided by the algorithm in two clusters, generating 3 clusters in total with 30, 25 and 40 samples respectively (*K* = 3). Following iterations keep dividing and merging the data set, up to the imposed limit of level 9 and *K* = 10, where the clusters formed had few samples and a quite small separation distance, making further iterations pointless in terms of the subsequent classification stage.

The *x* axis shows the color coded labels of the ectopic foci grouped for *K* = 10 (i.e., level 9 and last iteration). It is important to recall that a particular ectopic focus should be the same for any of the 5 different distributions of fibrosis (i.e., Case 1 to Case 5). The groups formed are shown separated by a thick black vertical line. At this hierarchy level, the simulations for ectopic focus LA5 (simulated 5 times for M1, corresponding to Case 1 to Case 5) did not fall in the same cluster, but 3 BSPiMs were classified into Class 3, and 2 BSPiMs to Class 4 (see Figure 6, encircled labels). This fact indicates how the presence of fibrosis, already in Model M1, starts to affect the BSPiM profiles, and consequently the clustering process.

The clustering of the BSPiM maps obtained above corresponds to groups formed by ectopic locations on the atria. When we choose *K* = 2, for example, all ectopics were arranged into cluster 1 or 2, if we chose *K* = 6, all ectopics were then distributed into clusters 1–6.

#### 3.2.2. Association of BSPiM clusters to regions on the atria

After clustering the BSPiM maps, we associated to each cluster the ectopic beats that generated the corresponding BSPiM in the cluster. Next, for each cluster, we summed the areas on the atria surface that were linked to each ectopic beat, i.e., the ectopic patches. We obtained this measure for *K* = 2 to *K* = 10. For example in the case of *K* = 2 we had the area of cluster 1 and cluster 2, and then calculated the mean area, and the standard deviation in the areas, to obtain a single representative measure of the atrial regions. Note that if a given patch in the atria had two labels (it was expected to have a single label), its area would be summed twice to take into account the existence of region overlaps.

This measure is shown in Table 5 as $\hat{\text{X}}$ ± σ (cm^2^). The results show, as expected, a decrease in the mean and standard deviation of the region area as we incremented the number of clusters from *K* = 2 to *K* = 10 (row wise). When we analyzed those areas moving from M1 to M5 (column wise), there was an increase of the areas as the level of fibrosis increased. This finding is due to the region label overlap, since when a patch is labeled with more than one label it contributes to the sum of areas of more than one region. Therefore, the sum of all areas of all regions is larger than the area of the atria surface when there is label overlap. This increment is explained with another measure which is the ectopic persistence within the clustering process.

**Table 5:** Clustering performance results for the models M0–M5.

***K*_*i*_**	**Measure**	**M0**	**M1**	**M2**	**M3**	**M4**	**M5**
**K2**	$\hat{\text{X}}$ ±σ	66 ± 41	68 ± 39	78 ± 53	81 ± 49	68 ± 67	84 ± 44
	OR	–	–	10.53	15.79	–	21.05
	CR	100	100	100	100	100	100
	CA	0.98	1.00	1.00	0.99	0.97	0.91
**K4**	$\hat{\text{X}}$ ±σ	33 ± 14	34 ± 16	39 ± 22	40 ± 30	52 ± 28	61 ± 17
	OR	–	–	10.53	15.79	42.11	52.63
	CR	100	100	100	100	100	100
	CA	0.94	0.85	0.93	0.84	0.90	0.90
**K6**	$\hat{\text{X}}$ ±σ	22 ± 10	22 ± 13	27 ± 14	32 ± 20	42 ± 25	44 ± 25
	OR	–	–	15.79	36.84	63.16	68.42
	CR	100	89.4	100	94.7	94.7	94.7
	CA	0.91	1.00	0.96	0.97	0.96	0.93
**K8**	$\hat{\text{X}}$ ±σ	16 ± 9	17 ± 7	20 ± 13	27 ± 15	37 ± 20	43 ± 19
	OR	–	–	15.79	47.37	68.42	73.68
	CR	100	84.2	89.4	94.7	89.4	89.4
	CA	0.92	1.00	0.98	0.96	0.93	0.88
**K10**	$\hat{\text{X}}$ ±σ	13 ± 8	14 ± 8	17 ± 11	24 ± 15	32 ± 17	37 ± 17
	OR	–	5.26	21.05	52.63	78.95	78.95
	CR	100	78.9	84.2	89.4	89.4	89.4
	CA	0.85	1.00	0.98	0.96	0.89	0.88

*$\hat{\text{X}}$ ± σ: Mean Area ± Standard Deviation (cm^2^)*.

*OR: Overlapped ratio (%)*.

*CR: Connectivity ratio (%)*.

*CA: Classification Accuracy - Results for 256 characteristics*.

**Figure 7:**
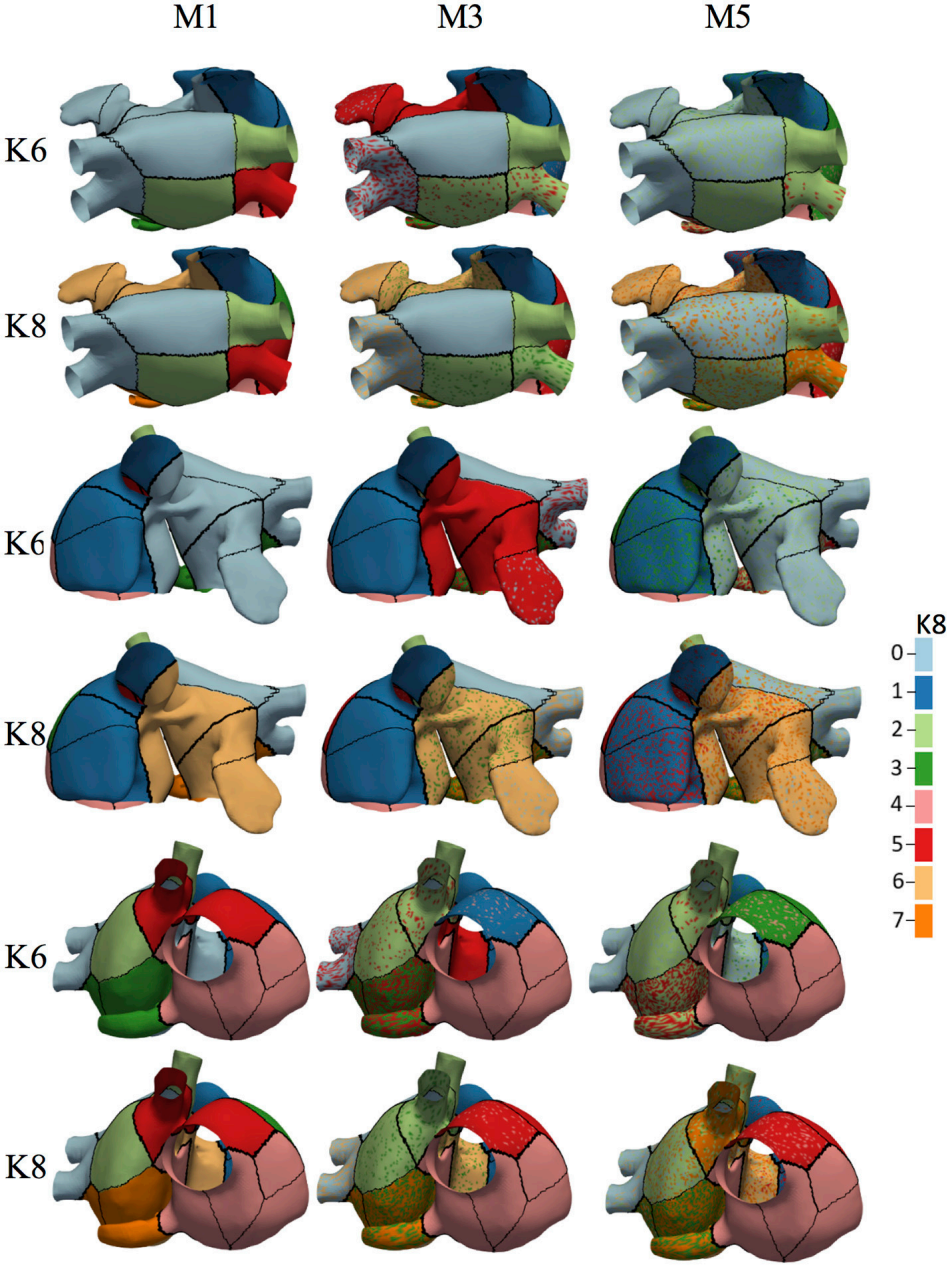
Associated BSPiM clusters for models M1, M3, and M5, for *K* = 6 and *K* = 8 (color coded) to the 19 patches defined by the atrial ectopic foci simulated with fibrosis (three different views). Regions including colored dots indicate that samples of a particular ectopic focus have spread into two or more clusters, and the corresponding atrial patches overlapped on the atrial surface exactly at that ectopic focus. Model M1 shows no overlapping for *K* = 6 and *K* = 10, and models M3 and M5 show an intensification of overlapping as the fibrosis stage increased. The color legend indicates the class/cluster number assigned to each atria patch.

Figure 7 shows the 19 atrial patches for the models M1, M3, and M5 ( an increasing level of fibrosis) color-coded with the assigned label for *K* = 6 and *K* = 8 (in three different atrial views). Patches with the same color form a unique region that relates to a group of BSPiM patterns. When a patch within a region shows more than one color or label ( a region with different colored dots), it means that two different BSPiM could be mapped to the same patch due to the variability introduced by the fibrosis. Therefore, patches colored as “red” and patches with “red dots” were all considered as a single region. However, our goal was to create regions that were as small as possible, in order to reduce the search area of the ectopic focus.

#### 3.2.3. Ectopic focus persistence within a clustering process

When models with fibrosis were included, they did not cluster properly into non-overlapped groups since BSPiM were highly altered by the conduction blocks. From stage 3 (i.e., 15% fibrosis) onwards the BSPiMs showed large differences for ectopic beats placed in the pulmonary veins, as can be seen in the example of Figure 5B, where the LATs of ectopic LA10, from Stage 3 to Stage 5 of fibrosis, show clear blockades at the pulmonary veins and left atrial appendage, and consequently completely different BSPiMs with a lesser level of fibrosis.

From the dendrogram of Figure 6 we observed that for the Model M1, when *K* = 10, due to the variability among these BSPiMs, we found that 3 of the LA5 BSPiMs were grouped into cluster 3 while the 2 remaining BSPiMs (with more fibrosis) were in cluster 4. This result indicates that samples of the ectopic LA5 have been spread into two clusters, so the corresponding atrial patches will overlap on the atria surface exactly on LA5. In Figure 7, ectopic foci locations with poor persistence values show regions that have spots of more than one color, indicating overlap. Figure S5 shows, as an example, the BSPiM together with their labels for model M3 and *K* = 6. A given ectopic focus can be classified into two different classes as a function of the fibrosis, e.g., class green and red for LA1.

For the results of the persistence analysis, we calculated the ratio of ectopics that appear in more than one cluster with respect to the total number of ectopic foci in the atria, (i.e., 57 ectopics for model M0, and 19 ectopics for models M1 to M5); Table 5 displays this measure as Overlapped Regions (OR).

The Model M0 (no fibrosis), showed, as expected, no overlap, given that there was no more than one label per ectopic focus (i.e., OR = 0). Results are summarized in Table 5. For the models with fibrosis, there was an increase of overlap as we incremented the level of fibrosis from M1 to M5 (column wise) and an increase of overlap as K increased from 2 to 5 (row wise), with the particular case of M1 with no overlap except for *K* = 10 with LA5 being spread in two clusters and OR = 5.26.

#### 3.2.4. Geometrical consistence of clusters

The analysis of the cluster connectivity using graphs permitted us to identify which clusters were not well formed, i.e., were not connected. We always preferred connected regions without holes or islands over the atria surface, otherwise when we associated an ectopic focus to a cluster, the cluster was scattered in several regions of the atria instead of having a connected and delimited one.

We calculated the ratio of well-formed clusters or connected clusters (i.e., well formed over all clusters) as the measure Connection Ratio (CR) from the validation process of the model M0 without fibrosis, and the five model configurations (M1–M5) with fibrosis, for K ranging from 2 to 10 clusters. Results are summarized in the Table 5.

For the Model M0 (no fibrosis), from *K* = 2 to 10, the Connection Ratio (CR) was 100%. The results for the model M1 (with fibrosis), for *K* = 2 and *K* = 4 show that the connection ratio was 100% and maintained with all models and levels of fibrosis (column wise). When we increased the number of groups from *K* = 6 to *K* = 10 (row wise), but also with the increase of fibrosis from M1 to M5 (column wise) there was a variable decrease of the ratio CR, implying the loss of connection or isolation of some ectopics from the groups. This effect depended on the different configurations of the patchy fibrosis (see Table 5).

**Figure 8:**
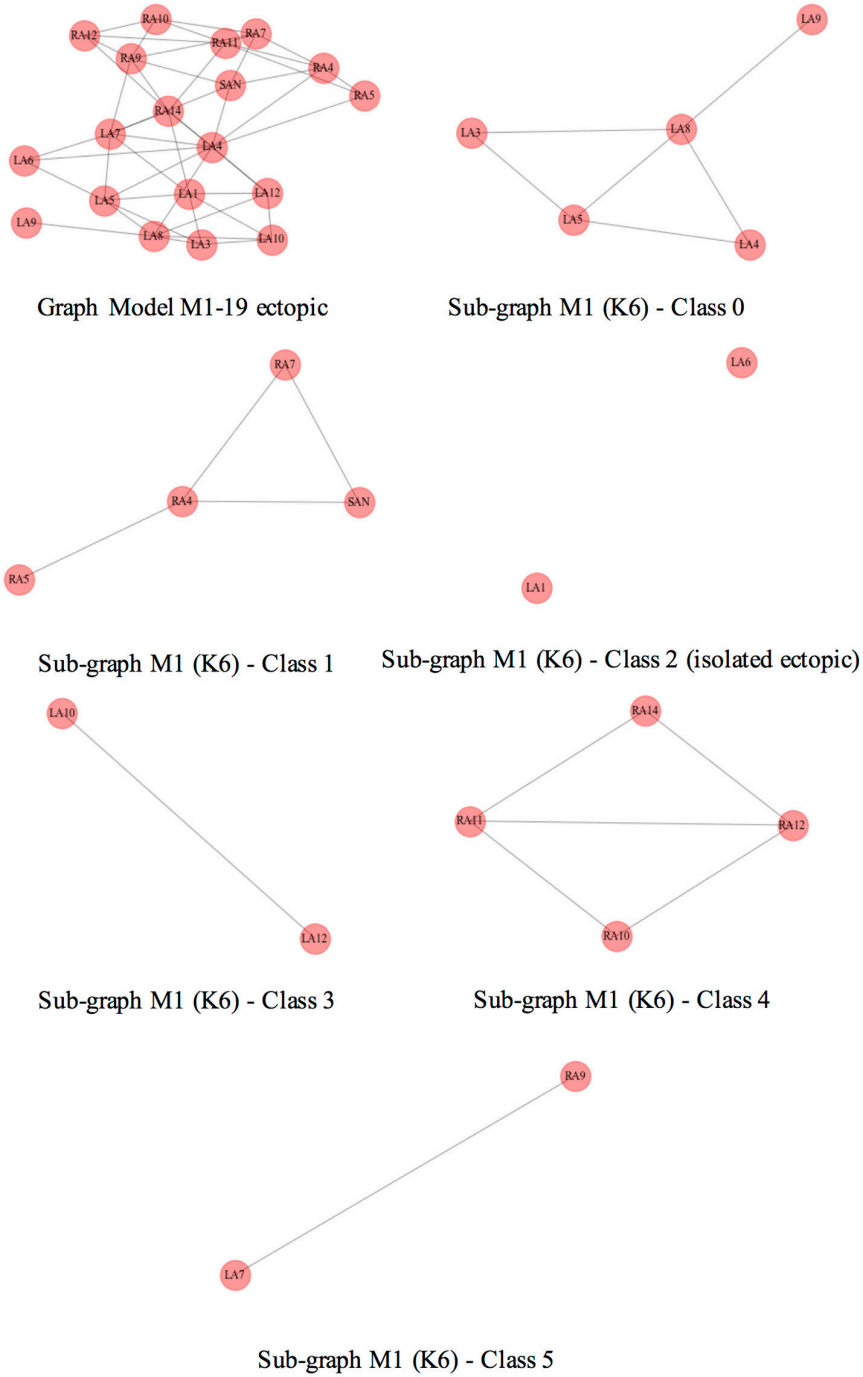
Geometrical consistence of clusters. Top row, on the left, shows the complete graph of the atria where the nodes represent the ectopic foci connected by edges. The rest of the figure shows all the subgraphs that represent the classes of the model M1 and *K* = 6. All of them are well formed connected subgraphs, except for the one at the middle row, on the right, representing ectopic foci LA1 and LA6 of the class 2 and indicating no connected nodes.

As an example, the Figure 8 depicts at the top row, on the left, the complete ectopic graph of the atria with the nodes representing the 19 ectopic foci connected by edges; the rest of the figure shows the 6 subgraphs of each class, formed after clustering the model M1 for *K* = 6. The subgraph in the middle row, on the right, shows the ectopic foci LA1 and LA6 with no connection, (i.e., 2 isolated nodes with respect to 19 total nodes, therefore a CR of 89.4%).

We observed that the isolated ectopics for the particular case of model M1 were mainly the LA1 and LA5, both located at the center of the posterior wall of the left atrium, lower and upper regions, respectively, and LA6 at the upper Right pulmonary vein. More ectopics were located in the right atrium, RA7 and RA11, right above the pectinate muscles. In the case of the model M2, the isolated ectopics were the same as in model M1, except for LA1; for the models M3 to M5, the isolated ectopics were LA7, at the lower right pulmonary vein, and LA9 at the left appendage.

### 3.3. Classification of atrial ectopic focus

**Figure 9:**
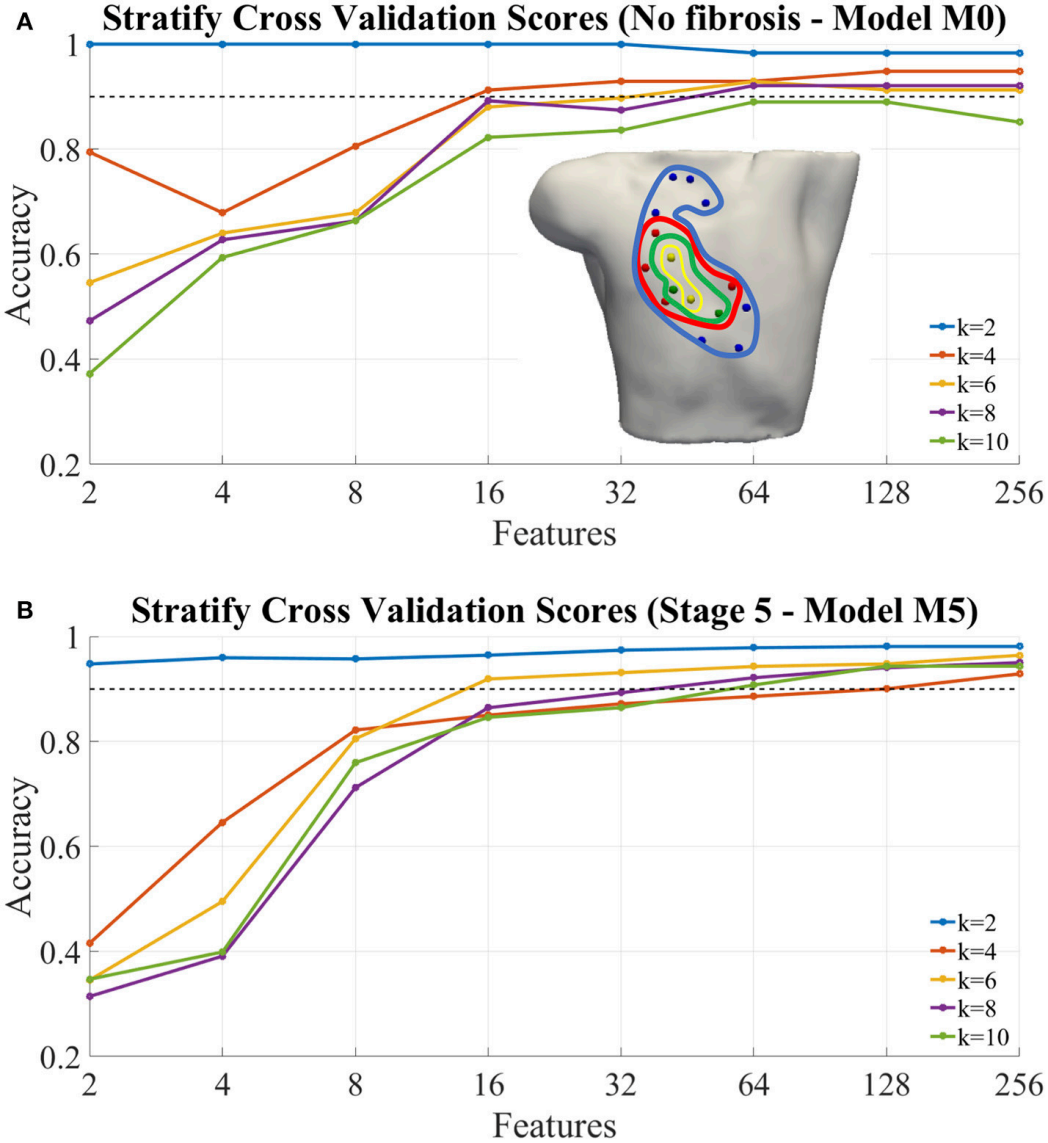
Classification of ectopic foci. **(A)** Accuracy of classification for the model M0, no fibrosis; **(B)** Accuracy of classification for the model M5, extreme case of fibrosis (i.e., from Stage1 to Stage 5). In both cases, the graphs were performed for different number of ectopic clusters (from *K* = 2 to *K* = 10), and different number N of features used (i.e., 2, 4, 8, 16, 32, 64, 128, and 256). Inset **(A)**, example of optimal selected features (electrodes) for: 2 (yellow), 4 (+green), 8 (+red), 16 (+blue) features.

After the analysis of the persistence and geometrical consistence of the clustering process, we performed a classification of the ectopic foci, according to the step 5 of our pipeline. The Figure 9 gives an example of the calculation of the accuracy for the model M0, no-fibrosis, and the extreme case of the model M5 that includes all the stages of fibrosis. The values obtained came from the cross-validation process which averages the accuracy obtained for each fold and summarizes the result. We used 4-folds to split and stratify homogeneously the training set and the test set. We have also included in the plot a dotted line (accuracy = 0.90) that is the minimum level of accuracy that we considered necessary to use the model in a clinical environment.

#### 3.3.1. Classification of non-fibrotic cases

Table 5 shows the measured classification accuracy (CA) for 256 features for the model M0 (i.e., no-fibrosis) and for *K* = 2 to *K* = 10. The results show that for this case of no-fibrosis we obtained an accuracy CA > 0.90 for *K* = 2 to *K* = 8, and a minimum accuracy CA = 0.85 for *K* = 10. Figure 9A, shows the complete accuracy obtained by the classification of the model M0 for features or electrodes from 2 to 256. In the cases *K* = 2 to *K* = 8 we recorded an accuracy of CA > 0.90 even with only 64 features or electrodes, and for *K* = 10 the accuracy was entirely below the dotted line of CA = 0.90. Inset Figure 9A, shows a torso with an example of the optimal electrode locations selected from the 256-electrode BSPiM to perform the ectopic foci classification in groups. For example, yellow spheres correspond to the best set of 2 electrodes, whereas yellow together with green spheres correspond to the best set of 4 electrodes. Larger sets of optimal electrodes always contained smaller sets. There were no intersecting classes, and all the ectopic groups were associated to different regions.

#### 3.3.2. Classification of fibrotic cases

We introduced incrementally in the training phase for classification those fibrotic cases from model M1 to model M5 (i.e., data from all FAT simulations, and all configurations of fibrosis). Table 5, shows the measured CA for the models M1 to M5 for *K* = 2 to *K* = 10 and 256 features, where the values of accuracy remained almost all above CA > 0.90 for all the models with slight variations, except for model M3 and *K* = 4, that showed a minimum of CA = 0.84.

Figure 9B shows the complete accuracy obtained for the extreme case of M5 (i.e., Stage 5 of fibrosis). We see, in this case, from *K* = 2 to *K* = 6 that we need a minimum of 16 features to obtain an accuracy CA = 0.90, and a maximum of CA = 0.93 with 256 features for *K* = 6. From *K* = 8 to *K* = 10 the values of accuracy are below the dotted line of 0.90, obtaining values of CA = 0.8 for both, *K* = 8 and *K* = 10. Figure S6 includes accuracy plots for all the models, M0–M5.

## 4. Discussion

### 4.1. Coupling atrial myocytes and fibroblast

Several modeling studies have illustrated the impact of fibrosis on atrial electrophysiology and conduction as well as on ECGs and showed in a realistic atrial anatomy that increased anisotropy in the atria due to fibrosis can be responsible for the breakup of PV ectopic waves into multiple re-entrant circuits. Maleckar et al. (2009) coupled a human atrial myocyte to a variable number of fibroblasts and investigated the effect of altering the intercellular coupling conductance, electrophysiological fibroblast properties, and stimulation rate on the atrial AP. The results demonstrated that the myocyte resting potential and AP waveform are modulated strongly by the properties and number of coupled fibroblasts, the degree of coupling, and the pacing frequency. Jacquemet et al. developed a 2D model of atrial tissue including microfibrosis incorporated as a set of thin collagenous septa (sheets) of cardiac muscle to determine whether they, like thick collagenous septa, could affect electrical impulse propagation and disconnect transverse coupling (Jacquemet, 2012). The density and length of these septa were varied and the analysis of unipolar electrograms showed that the septa decreased conduction velocity (CV) by up to 75%. Another important aspect to be considered is the existence of collagen layers in the fibrosis model. Atrial models incorporating transverse collagen deposition have underlined the significant interruption and disorder in atrial conduction patterns (Boyle et al., 2016). Not only the total amount of collagen was important, but also the specific spatial distribution of collagen deposition, which governed the occurrences of conduction block. Another novel arrhythmic mechanism being considered in models is percolation (slow and difficult fluid flow through a porous medium). It has been shown that simulation of conduction obstacles derived from LGE-MR images of AF patient atria, give rise to excitation patterns resembling near-threshold percolation (Vigmond et al., 2016). In this context, the percolation threshold is the fraction of lattice points that must be filled to create a continuous path of nearest neighbors from one side to another.

In our study, we evaluated the degree to which coupling fibroblasts to atrial myocytes altered the electrophysiology of the normal myocytes. Our simulations confirmed that the coupling of fibroblasts to myocytes significantly affects the electrophysiological properties of the myocytes, as described by MacCannell et al. (2007), Maleckar et al. (2009), and Morgan et al. (2016).

The coupling of the CRN (Courtemanche et al., 1998) atrial myocyte model to the active formulation of the MacCannell fibroblast model (i.e., 4 membrane currents including, the time and voltage dependent fibroblast current *I*_*Kv*_, the inward rectifying current *I*_*K*1_, the *Na*^+^-*K*^+^ pump current *I*_*NaK*_, and the background *Na*^+^ current *I*_*b, Na*_) (MacCannell et al., 2007), and the cell-to-cell electrotonic interaction, caused: (i) a reduction of myocyte APD; (ii) a prolonged repolarization of the AP compared to the uncoupled myocyte control model AP; and importantly since fibroblasts have a higher resting membrane potential (RMP), (iii) changes of the myocyte RMP, see Figure 4B.

Furthermore, this shortening of the APD generates a spatial heterogeneity within the atrial tissue due to variations in the fibroblast density and the number of coupled fibroblasts to myocytes, generating a variation of the APD that depends, to a great extent, on the point where the measurement is taken in our virtual human atrial mesh. Although there were variations in the *APDs*_90_, dependent on the test location, density of fibrosis, and the number of coupled fibroblast to that point, all the APDs were shorter than the uncoupled myocyte control case, see Figure 4B.

### 4.2. Ectopic foci localization

Computational modeling of the human atria has changed during the last 15 years, evolving from very simple structures to very detailed models including atrial wall and fiber directions (Doessel et al., 2012). Several models exist today that include structures of intracellular compartments and atrial heterogeneity, and furthermore they include pathological structures, modeling atrial remodeling and fibrotic tissue.

MacLeod et al. (2008) emphasized the importance of including information about structural changes of the atrial myocardium into geometrical models. Previous results from Kistler et al. (2006) suggest that FATs have a particular electrical pattern on the torso (Morton et al., 2001; Kistler et al., 2003a,b, 2005), and that those patterns have a singular P wave morphology in specific locations providing a potential way to predict the origin of FATs. They developed a decision tree algorithm based on the P-wave morphology in specific surface ECG leads to provide some help in the search for ectopic foci sources to allow for the identification of the origin of the tachycardia. Therefore, using only the P wave morphology, they prospectively evaluated the algorithm with a number of patients, finding a predictive accuracy of 93% for a few focal trigger locations that could be distinguishable.

Other biomarkers such as P-wave integral maps (SippensGroenewegen et al., 1998) have been recommended to summarize different atrial activation sequences and relate them to ectopic foci. In the ventricles, other complex techniques such as electrocardiographic imaging (ECGi), have been widely studied in the last few decades to directly compute the cardiac action potentials by solving an ill-posed inverse problem (Ramanathan et al., 2003; Van Oosterom, 2012). However, many of those approaches use a priori information to improve their results, such as constraints in spatial and temporal domains, physiological knowledge about the activation sequence or localization of activation onset. In addition, those methods need a segmentation of the atria and torso models from an image sequence stack, and the construction of a finite element model to simulate cardiac electrophysiology. All those requirements, which are very time consuming, hamper the use of those tools in clinical environments.

In our previous work Ferrer-Albero et al. (2017), we used machine learning techniques to spatially cluster and classify ectopic atrial foci into clearly differentiated atrial regions by using the body surface P-wave integral map (BSPiM) as a biomarker. Ectopic foci with similar BSPiM naturally clustered into differentiated, non-intersected atrial regions and new patterns could be correctly classified with an accuracy of 97% when considering 2 clusters and 96% for 4 clusters (Ferrer-Albero et al., 2017). However, we only considered non-fibrotic cases, which are not very common cases clinically.

To learn this association, (i.e., ectopic location-BSPiM), regression techniques could appear to be a reasonable approach. However, as the total number of ectopic locations is reduced (i.e., 57 = 19 with fibrosis + 38 without fibrosis), there are not enough ectopic locations to apply regression techniques.

In this multi-scale biophysical 3D model simulation study, we used machine learning techniques to focus also on the localization of the arrhythmogenic electrical drivers (i.e., ectopic foci), that contribute to the generation of focal atrial tachycardia (FAT) with regional LA patchy fibrosis as a variable of structural remodeling according to the Utah classification scale (Oakes et al., 2009). This study introduces a new methodology which improves previous results and obtains an accuracy above 90% for classifying ectopics into 6 different atrial regions (i.e., from *K* = 2 to *K* = 6). In addition, we reduced the dimensionality of the BSPiM patterns and included noise to obtain data similar to that acquired in a clinical environment. It is important to remark that our simulated P-waves do not include QRS complex and are not affected by baseline wandering. In a real scenario, it will be fundamental to use filters such as bidirectional high-pass Butterworth filter to correct baseline wandering, or Template Matching Subtraction to eliminate the QRS complex. Feature selection analysis was used to find the minimum number of electrodes required to predict, with high accuracy, the location of ectopic foci during FAT. For cases without fibrosis, we could obtain predictions (dividing the atria in *K* = 4 regions) with an accuracy of 0.90 with only 16 features or electrodes placed on the torso front. When detection considered more and smaller regions (from *K* = 6 to *K* = 10), the accuracy was reduced to a minimum accuracy of 0.81 for *K* = 10, and a maximum of 256 electrodes.

As soon as LA patchy fibrosis comes into play, (i.e., Stage 1 to Stage 5), together with an increase in the number of regions analyzed, (i.e., *K* = 2 to *K* = 10), the measure of overlapped regions ratio increases, confirming that overlapping (see Figure S6), and the ratio of well formed clusters, or convexity, decreases, demonstrating the presence of ectopics disconnected or isolated from their group. However, the classification accuracy, remained above the value of 0.90 for numbers electrodes ranging from 128 to 256, even for the most extreme case, which is the model M5. The high accuracy was obtained because we allowed the clusters of patches to be disconnected. Therefore, if the model predicts that a given BSPiM relates to class n, the patches that form the class could belong to more than one single atrial region. From a clinical point of view, the location of the atrial trigger will not be so efficient since the area of search increases, but still the method improves current clinical practice. A positive point is that in cases in which a given atrial patch has more than one label, the main patches associated with the label are in general neighbors. That means that patches in the borders of two regions sometimes are classified as label “a” and sometimes a neighboring region “b.”

### 4.3. Study limitations

There are several limitations of the proposed methodology that need to be acknowledged. The most important is that although the activation patterns were validated against a clinical database, they have been simulated and do not correspond to real patients. In addition, patient atrial shape variability could introduce slight differences in the P wave morphology that in turn will affect the BSPiM patterns in some cases. Finally, the localization of ectopic focus is based on regions, and therefore the electrophysiologist still has to determine where exactly the focal point is within the predicted region.

## 5. Conclusions

The methodology presented here could be useful to help an electrophysiologist to reduce the search area of an ectopic focus non-invasively and plan the intervention a priori. The pipeline presented can produce results in real time, since all the simulations and the training phase are performed offline and a priori. The effect of fibrosis on the atrial activation and BSPiM is large when stage 3 (>15% fibrosis) is used. The machine learning system obtains high accuracy at the expense of increasing the size of the region where the ectopic focus is located. The most complex locations determined in our simulation study were in certain pulmonary veins when the stages of fibrosis were 3, 4, or 5. However, patients that show a stage of fibrosis higher than 3 are not recommended for treatment.

## Author contributions

All authors have made substantial contributions to this study. EG and RS conceived and designed the study, and drafted the manuscript, EG performed the investigation and simulation work. EG, ML, and RS contributed to data analysis, computer simulations and the software development. IG-F, AF-A, RM and JS contributed to the interpretation of the results and have revised the document critically. All authors have also approved the final version to be published while agreeing to be accountable for all aspects of the work in ensuring that questions related to the accuracy or integrity of any part of the work are appropriately investigated and resolved.

### Conflict of interest statement

The authors declare that the research was conducted in the absence of any commercial or financial relationships that could be construed as a potential conflict of interest.
