# Supplementary material for: Atrial Fibrosis Hampers Non-invasive Localization of Atrial Ectopic Foci From Multi-Electrode Signals: A 3D Simulation Study
Source: Front Physiol. 2018 May 18;9:404. doi: 10.3389/fphys.2018.00404 (PMC5968126; doi:10.3389/fphys.2018.00404)
Supplement: Supplementary file 1 [file Table_1.PDF]

Table S1: Right and Left atria anatomical regions and labels of simulated ectopic sites

Right Atria anatomical locations	10 – Labels of ectopic simulated with fibrosis & experimentally determined	31 – Labels of ectopic simulated with no fibrosis (additional ectopic sites)
Sinoatrial node	SAN	SAN
Crista Terminalis	-	R18
Intercaval Bundle	RA9	RA9, RA22, RA24, RA30
Right Septum	-	RA1, RA15, RA26
Right Wall	RA4, RA6, RA7, RA10, RA11, RA12	RA4, RA6, RA7, RA8, RA10, RA11, RA12, RA19, RA20, RA23, RA25
Right Appendage	RA5	RA5, RA21, RA27
Isthmus	RA14	RA14, RA28
Superior Cava Vein	-	RA2, RA3
Tricuspid Valve	-	RA13, RA16, RA17, RA29
**Left Atria anatomical locations**	**11 - Labels of ectopic simulated with fibrosis & experimentally determined**	**27 - Labels of ectopic simulated with no fibrosis (additional ectopic sites)**
Left Bachmann Bundle	LA4, LA8	LA4, LA8, LA22
Left Superior Wall	-	LA26, LA27
Left Septum	-	LA14
Left Appendage	LA9	LA9, LA19
Posterior Wall	LA1, LA5	LA1, LA5, LA16, LA17, LA18, LA20, LA23, LA24, LA25
Mitral Valve	LA10, LA11, LA12	LA10, LA11, LA12, LA13
Right Pulmonary Veins	LA6, LA7	LA6, LA7, LA21
Left Pulmonary Veins	LA3	LA2, LA3, LA15
