# Supplementary material for: Atrial Fibrosis Hampers Non-invasive Localization of Atrial Ectopic Foci From Multi-Electrode Signals: A 3D Simulation Study
Source: Front Physiol. 2018 May 18;9:404. doi: 10.3389/fphys.2018.00404 (PMC5968126; doi:10.3389/fphys.2018.00404)
Supplement: Supplementary file 2 [file Image_1.PDF]

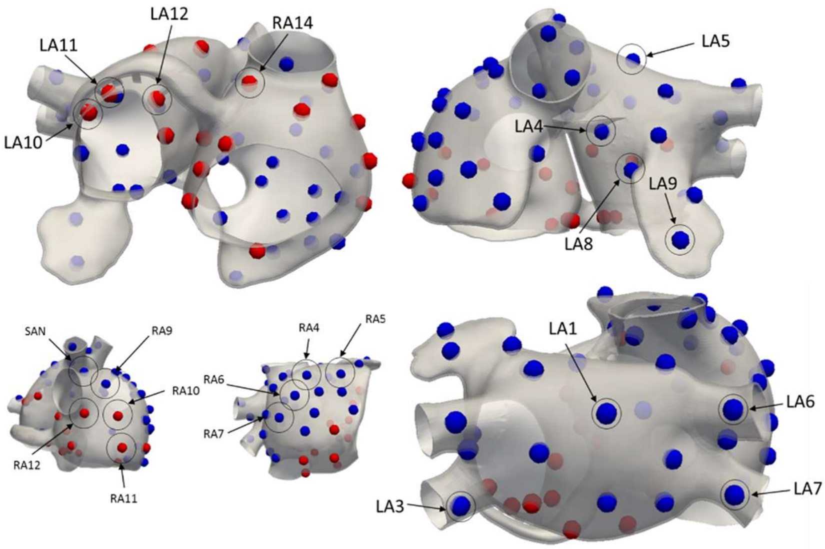

Figure S1: Ectopic location on the Right and Left atria. Ectopic foci without labels correspond to the additional ectopic sites simulated without fibrosis.
