# Supplementary material for: Atrial Fibrosis Hampers Non-invasive Localization of Atrial Ectopic Foci From Multi-Electrode Signals: A 3D Simulation Study
Source: Front Physiol. 2018 May 18;9:404. doi: 10.3389/fphys.2018.00404 (PMC5968126; doi:10.3389/fphys.2018.00404)
Supplement: Supplementary file 3 [file Image_2.PDF]

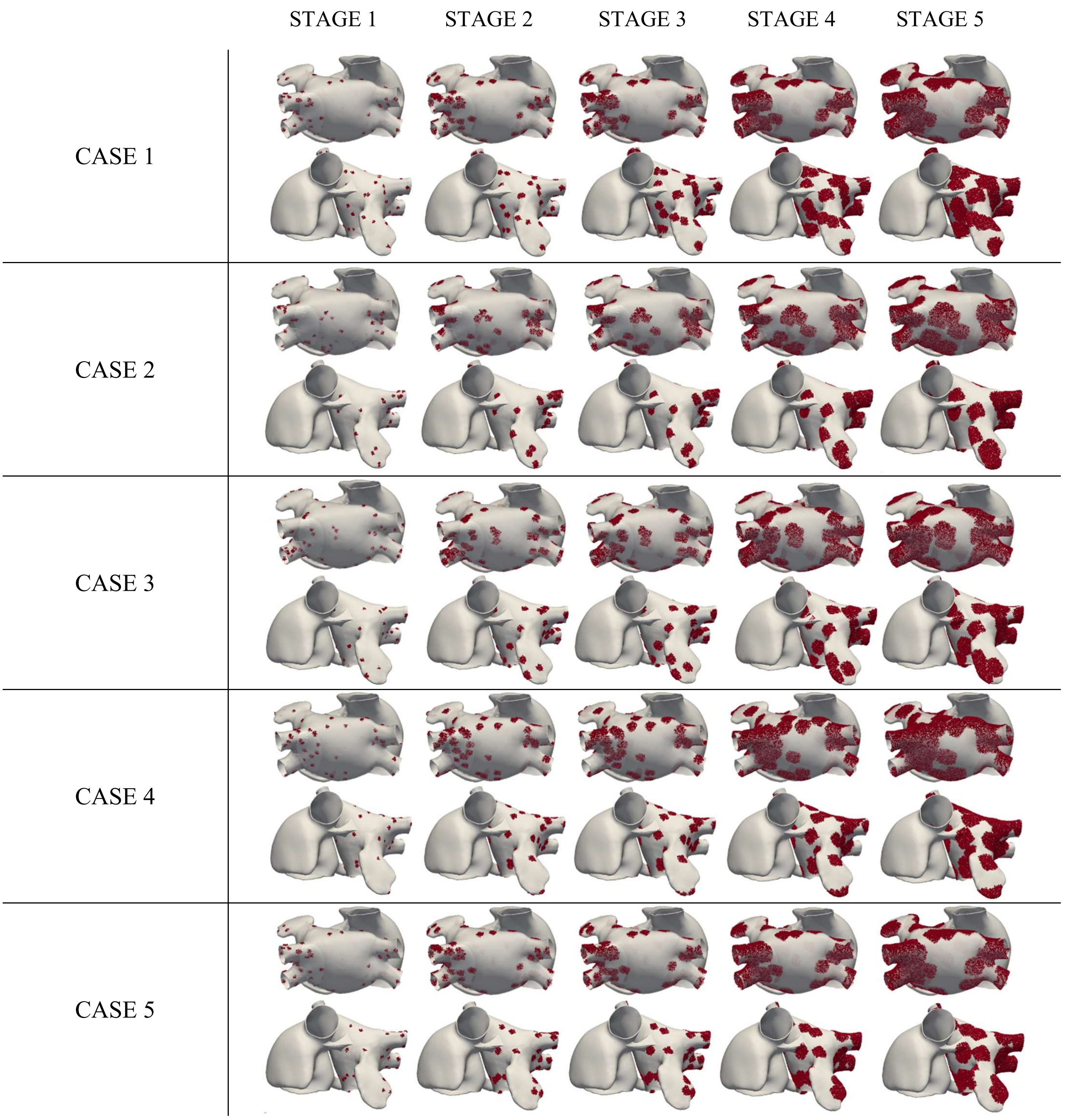

Figure S2: Complete set of 5 distributions of patchy fibrosis in 5 different proportions (stages) shown in the 3D atria in two different views. The stages were defined considering the Utah classification, (Utah stage I: < 8.1% (Q1); Utah Stage II: < 16% (Q2); Utah Stage III: < 21% (Q3); Utah Stage IV: > 21% (Q4)). Stage 5 corresponds to a higher density within the Utah Stage IV.
