# Supplementary material for: Atrial Fibrosis Hampers Non-invasive Localization of Atrial Ectopic Foci From Multi-Electrode Signals: A 3D Simulation Study
Source: Front Physiol. 2018 May 18;9:404. doi: 10.3389/fphys.2018.00404 (PMC5968126; doi:10.3389/fphys.2018.00404)
Supplement: Supplementary file 4 [file Image_3.PDF]

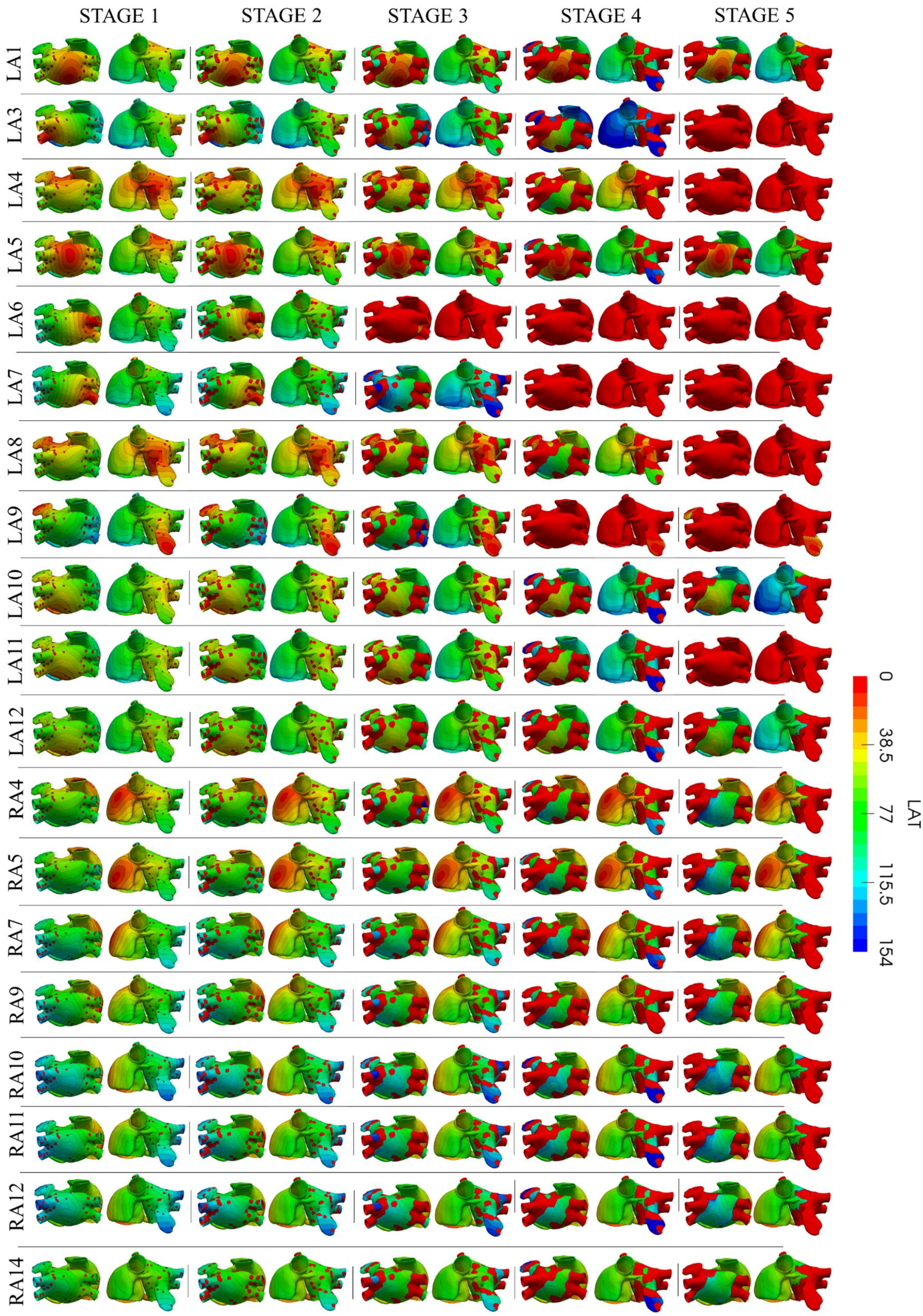

Figure S3: Local activation times for CASE 1, 19 ectopic, and STAGES of fibrosis 1 – 5. Isochrones are plotted every 10ms.
