# Supplementary material for: Atrial Fibrosis Hampers Non-invasive Localization of Atrial Ectopic Foci From Multi-Electrode Signals: A 3D Simulation Study
Source: Front Physiol. 2018 May 18;9:404. doi: 10.3389/fphys.2018.00404 (PMC5968126; doi:10.3389/fphys.2018.00404)
Supplement: Supplementary file 5 [file Image_4.PDF]

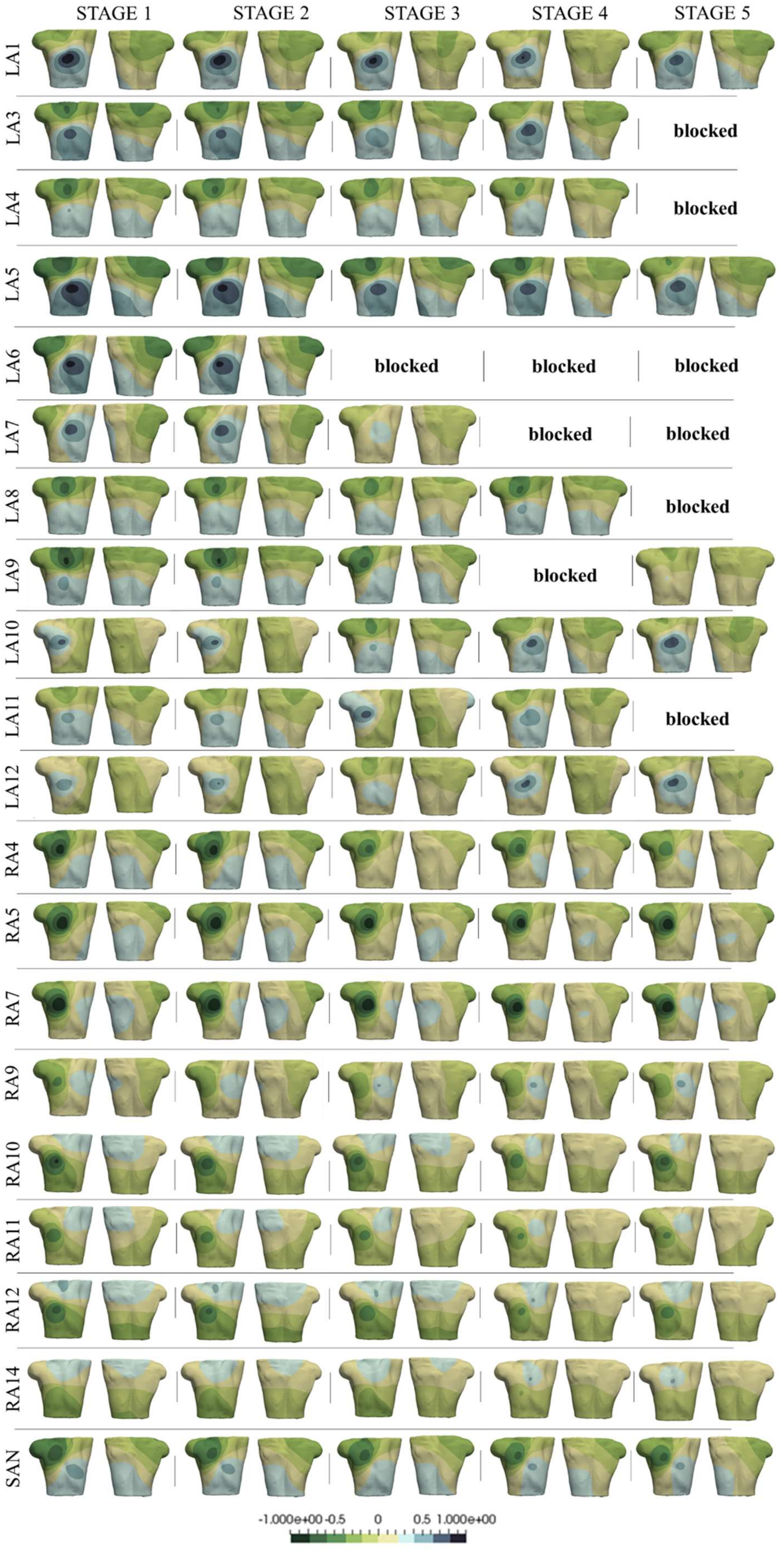

Figure S4: Corresponding BSPiMs generated for the 19 ectopic foci simulated with fibrosis (see Figure S2 above showing the LATs) for CASE 1 and STAGES 1 to 5.
