# Supplementary material for: Atrial Fibrosis Hampers Non-invasive Localization of Atrial Ectopic Foci From Multi-Electrode Signals: A 3D Simulation Study
Source: Front Physiol. 2018 May 18;9:404. doi: 10.3389/fphys.2018.00404 (PMC5968126; doi:10.3389/fphys.2018.00404)
Supplement: Supplementary file 6 [file Image_5.pdf]

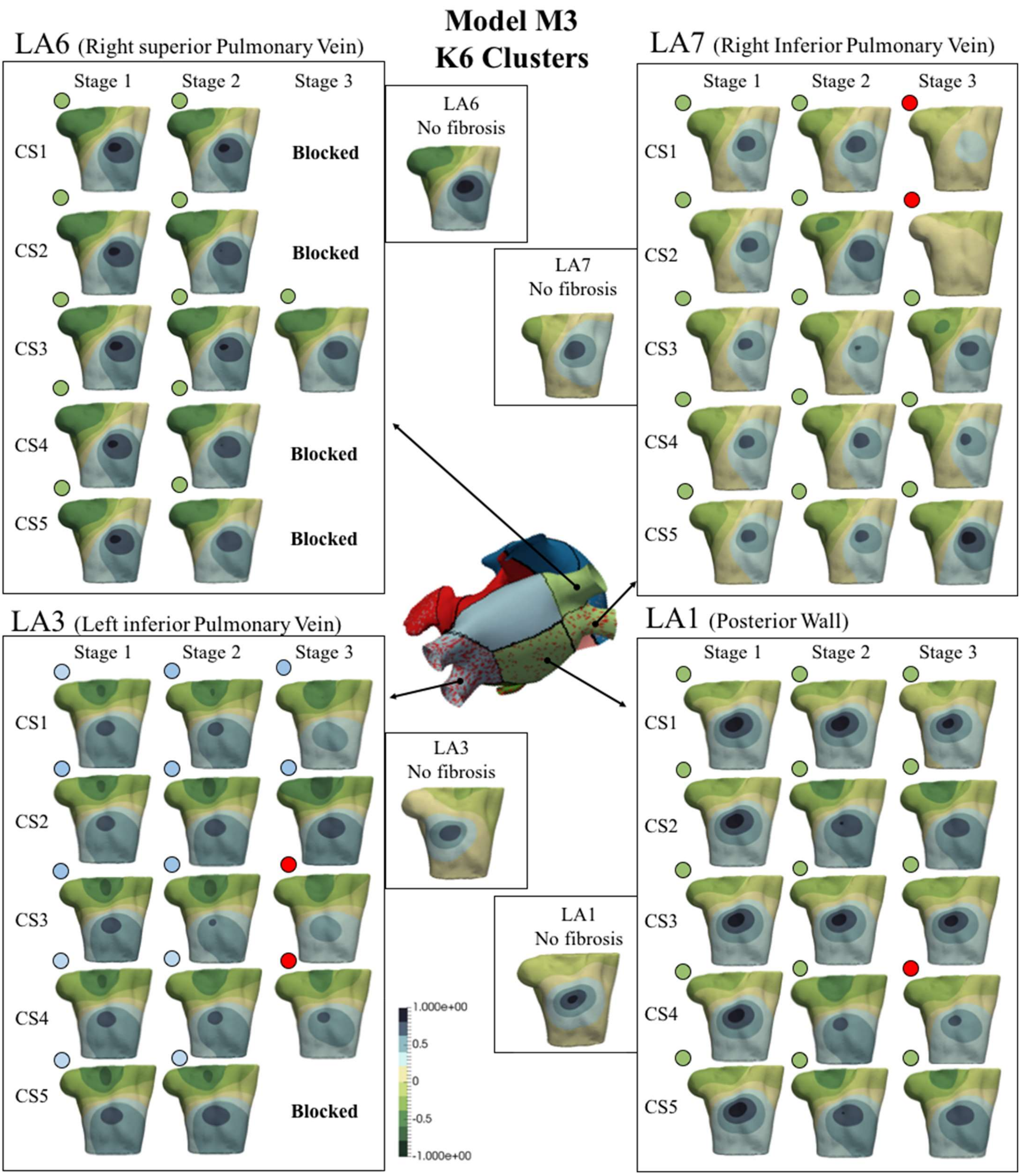

Figure S5: Atrial model color coded with the regions into which each patch has been classified. Classification corresponds to model M3 with K=6 regions. Patches with solid color are labeled into a single region, but dotted patches can belong to two or more classes. Detailed analysis of simulations for patches with more than one label shows that at Stage fibrosis 3, some BSPiM are classified into another class (red class in this case) for some patients. In this scenario, if a BSPiM is classified as red class, in addition to search for an ectopic focus in the left atrial appendage, there is a little chance that the ectopic focus is in LA1, LA3 or LA7, which increases the search area of the ectopic focus. That is why overlapped and/or unconnected regions are not desirable.
