# Supplementary material for: Atrial Fibrosis Hampers Non-invasive Localization of Atrial Ectopic Foci From Multi-Electrode Signals: A 3D Simulation Study
Source: Front Physiol. 2018 May 18;9:404. doi: 10.3389/fphys.2018.00404 (PMC5968126; doi:10.3389/fphys.2018.00404)
Supplement: Supplementary file 7 [file Image_6.PDF]

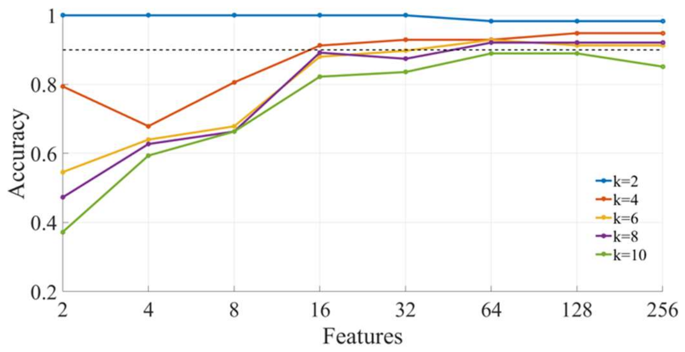

(A)

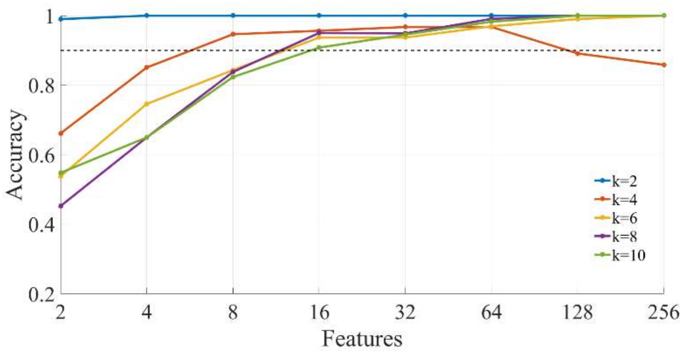

(B)

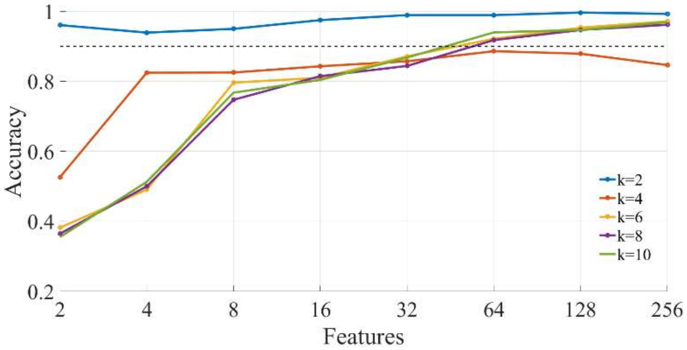

(C)

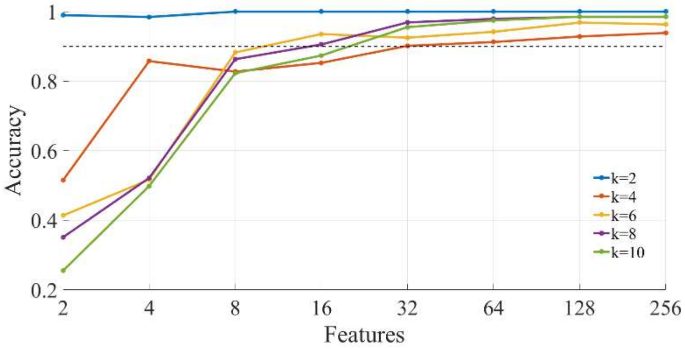

(D)

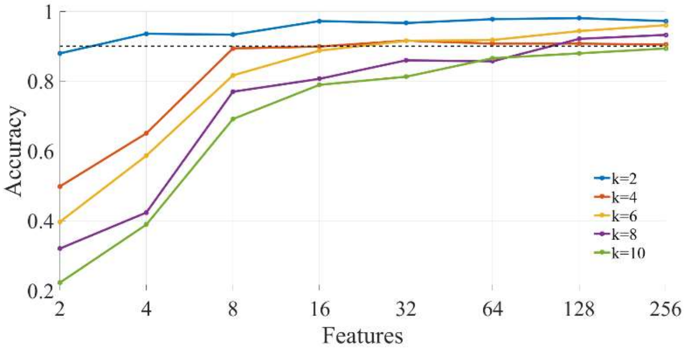

(E)

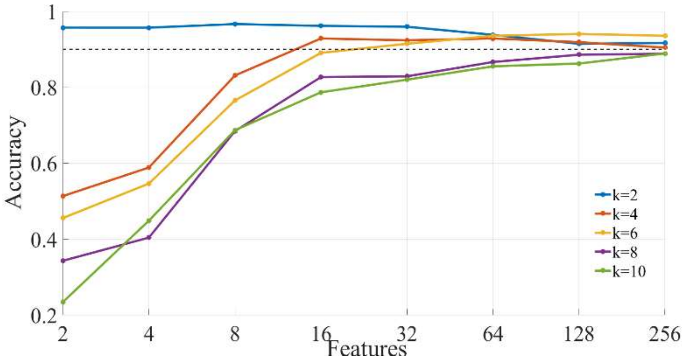

(F)

Figure S6: Complete set of the classification performance plots of ectopic foci. The graphs were performed for different number of ectopic clusters (from K = 2 to K = 10), and different number N of the features selected (i.e., 2, 4, 8, 16, 32, 64, 128, and 256 electrodes). (A) Accuracy of classification for the model M0, no fibrosis; (B), (C), (D), (E), and (F) Accuracies for the models M1 to M5, respectively.
